# Supplementary material for: Glycan reachability analysis: A bottleneck-aware framework for inferring tissue-specific glycan biosynthetic potential from transcriptomics
Source: PLoS Comput Biol. 2026 Sep 24;22(9):e1014655. doi: 10.1371/journal.pcbi.1014655 (PMC13606954; doi:10.1371/journal.pcbi.1014655)
Supplement: Supplementary file 1 [file pcbi.1014655.s001.pdf]

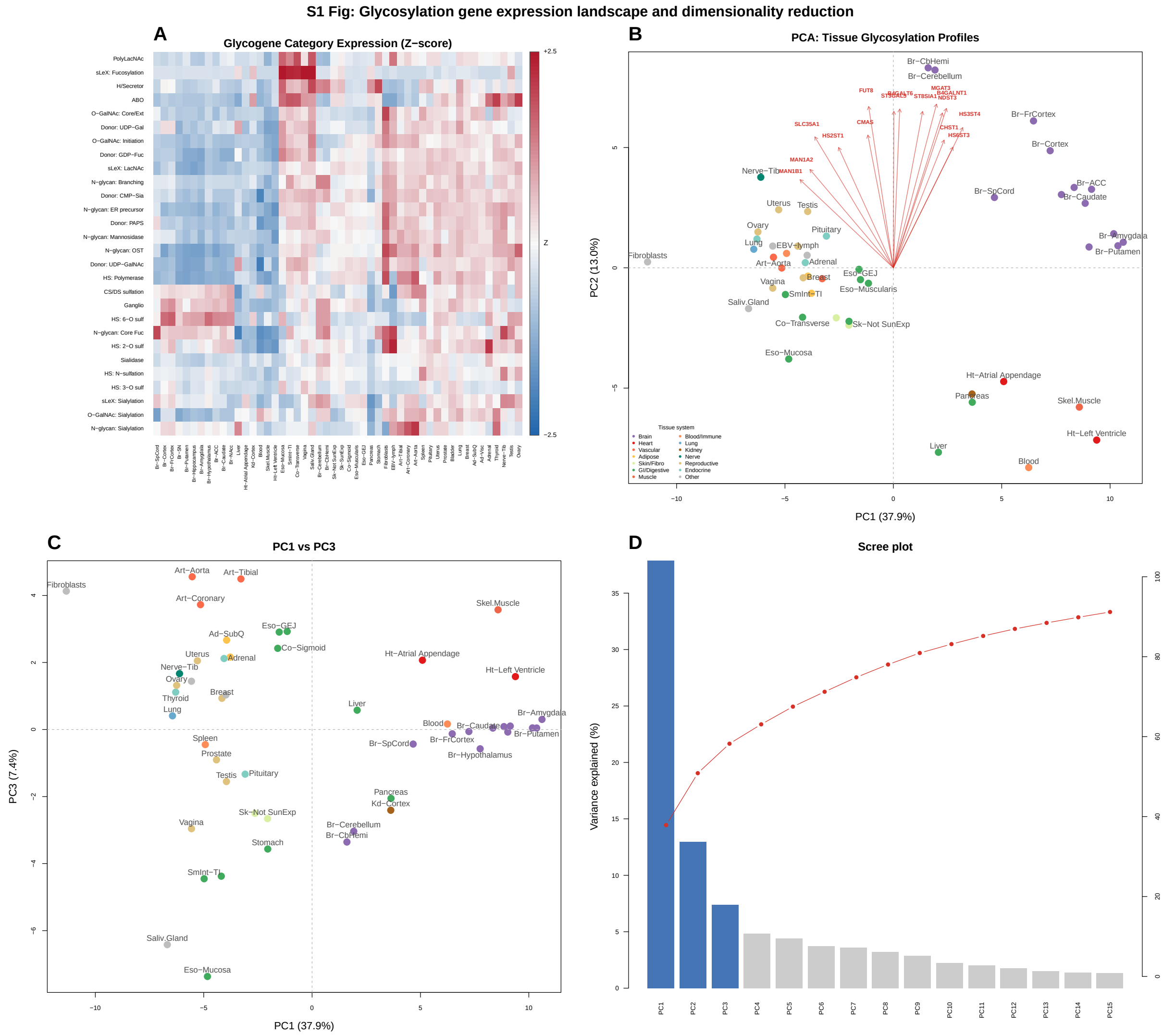

S1 Fig. Dimensionality reduction of glycogene expression. (A) Heatmap of median Z-scored glycogene expression across 50 GTEx tissues (rows: 25 gene categories; columns: tissues, hierarchically clustered). Brain tissues cluster separately with elevated ganglioside-related genes. **(B)** PCA biplot (PC1 vs PC2) of tissue-level glycogene expression profiles. Arrows indicate top gene loadings. **(C)** PC1 vs PC3, capturing additional variance along the HS/N-glycan axis. **(D)** Scree plot showing proportion of variance explained.
