# Supplementary material for: Glycan reachability analysis: A bottleneck-aware framework for inferring tissue-specific glycan biosynthetic potential from transcriptomics
Source: PLoS Comput Biol. 2026 Sep 24;22(9):e1014655. doi: 10.1371/journal.pcbi.1014655 (PMC13606954; doi:10.1371/journal.pcbi.1014655)
Supplement: Supplementary file 2 [file pcbi.1014655.s002.pdf]

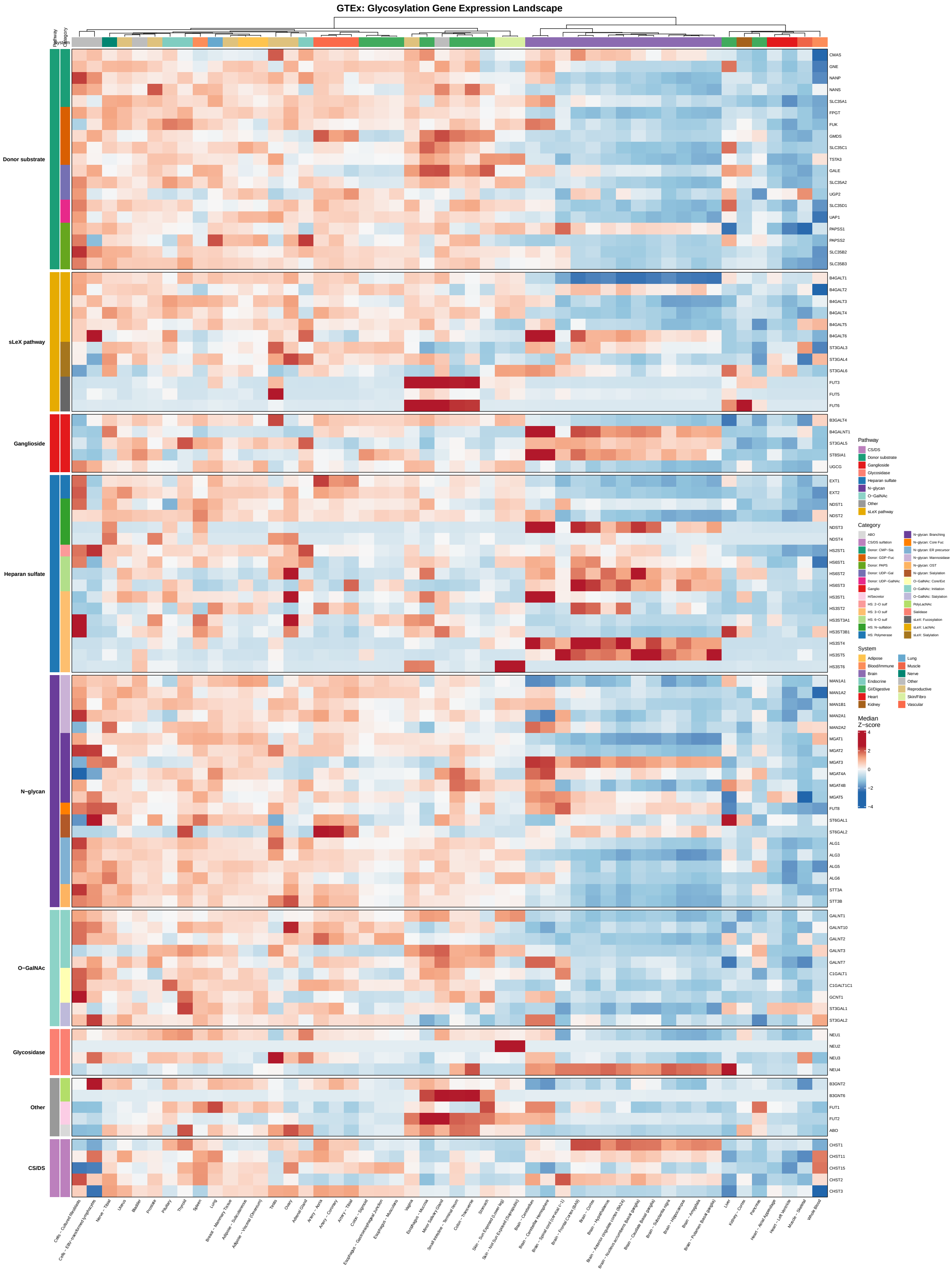

S2 Fig. Full glycogene expression landscape. Heatmap of median Z-scored expression for all 97 glycogenes (rows) across 50 GTEx tissues (columns). Genes are grouped by functional category (color sidebar); tissues are hierarchically clustered. Generated using ComplexHeatmap.
