# Supplementary material for: Glycan reachability analysis: A bottleneck-aware framework for inferring tissue-specific glycan biosynthetic potential from transcriptomics
Source: PLoS Comput Biol. 2026 Sep 24;22(9):e1014655. doi: 10.1371/journal.pcbi.1014655 (PMC13606954; doi:10.1371/journal.pcbi.1014655)
Supplement: Supplementary file 3 [file pcbi.1014655.s003.pdf]

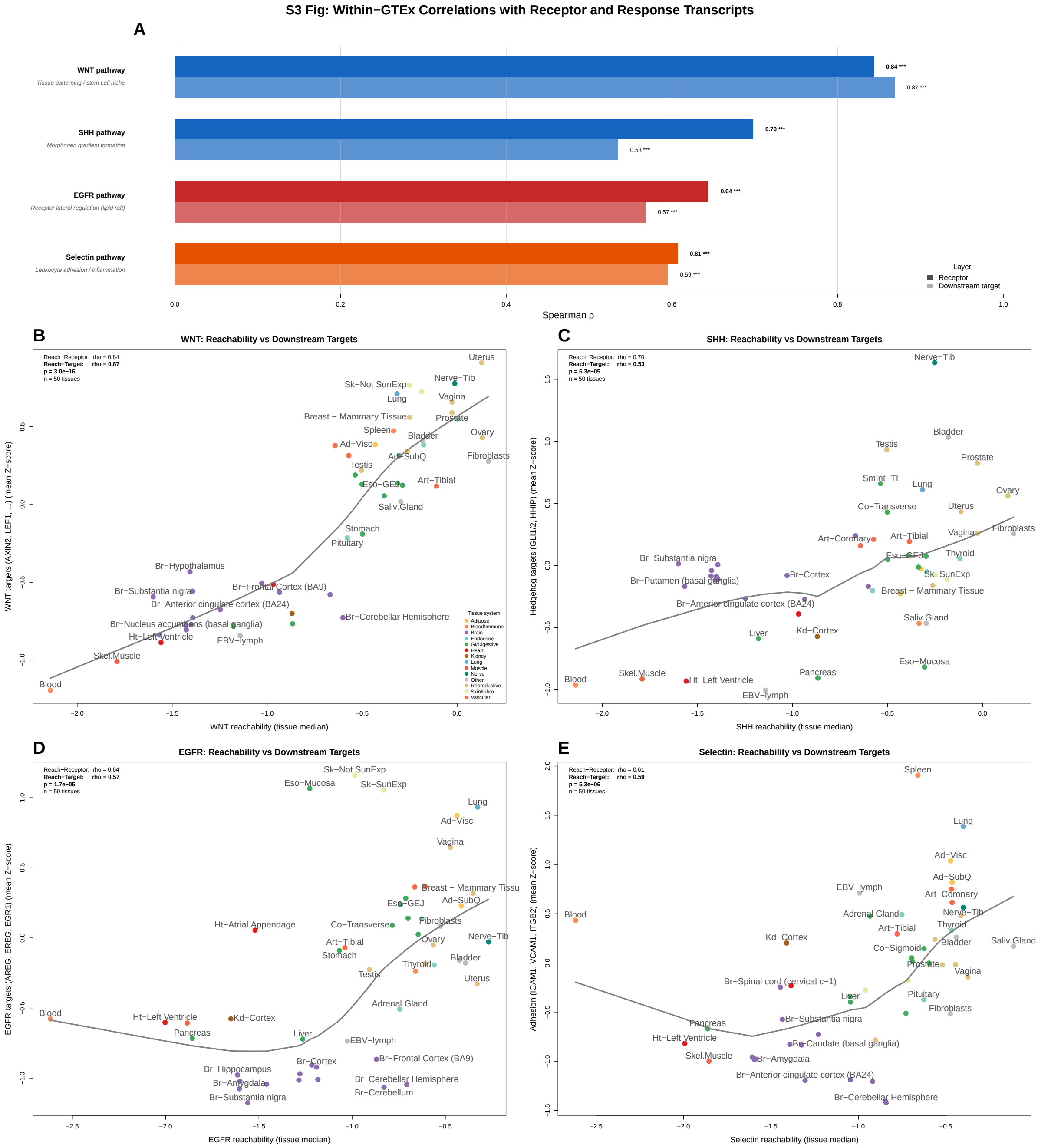

S3 Fig. Within-GTEx correlations with receptor and pathway-response transcripts. **(A)** Paired barplot of Spearman ρ for each cascade: reachability vs receptor (dark bars) and reachability vs downstream targets (light bars). **(B–E)** Tissue-level reachability versus response-transcript mean Z-score. Both axes derive from the same RNA-seq and do not establish glycan-mediated signaling.
