# Supplementary material for: Glycan reachability analysis: A bottleneck-aware framework for inferring tissue-specific glycan biosynthetic potential from transcriptomics
Source: PLoS Comput Biol. 2026 Sep 24;22(9):e1014655. doi: 10.1371/journal.pcbi.1014655 (PMC13606954; doi:10.1371/journal.pcbi.1014655)
Supplement: Supplementary file 4 [file pcbi.1014655.s004.pdf]

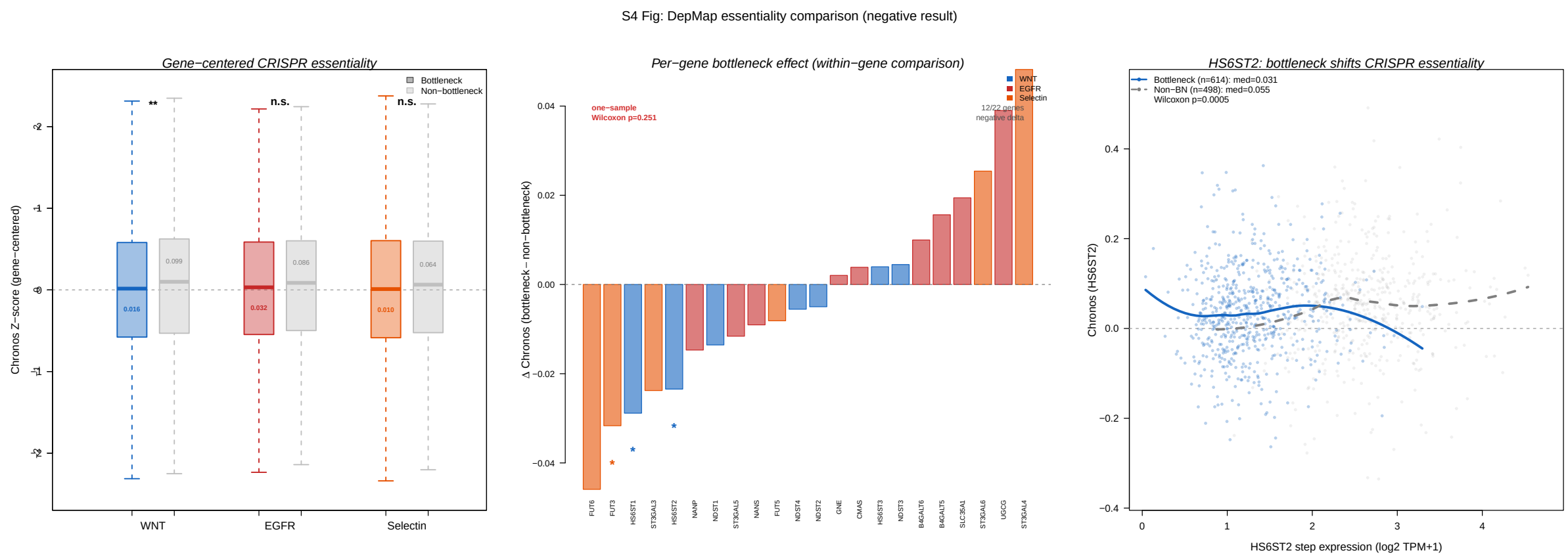

S4 Fig. DepMap essentiality analysis (negative result). Comparison of Chronos essentiality scores between cell lines where a glycosylation gene is an expression argmin versus elsewhere, for the three coherence cascades. Only HS6ST1 and HS6ST2 differ after BH correction; the overall result is negative.
