# Supplementary material for: Glycan reachability analysis: A bottleneck-aware framework for inferring tissue-specific glycan biosynthetic potential from transcriptomics
Source: PLoS Comput Biol. 2026 Sep 24;22(9):e1014655. doi: 10.1371/journal.pcbi.1014655 (PMC13606954; doi:10.1371/journal.pcbi.1014655)
Supplement: Supplementary file 5 [file pcbi.1014655.s005.pdf]

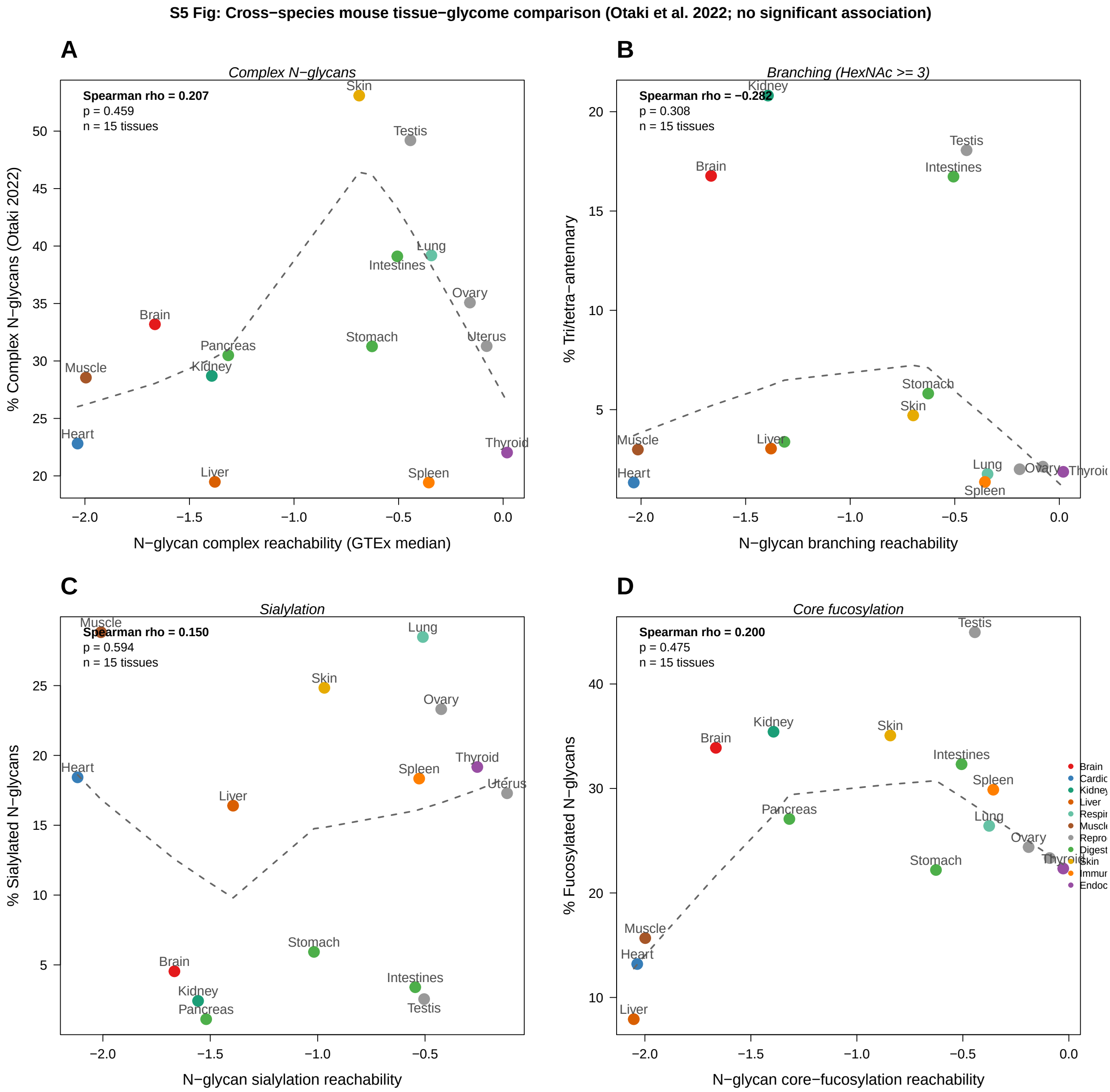

S5 Fig. Cross-species tissue glycomics comparison (negative result). Scatter plots of human GTEx-derived N-glycan reachability scores (x-axis) versus experimentally measured mouse tissue glycomic features (y-axis) from Otaki et al. [2] for $n = 15$ matched tissues, colored by organ system. **(A)** Complex N-glycans ($\rho = 0.207$, $p = 0.459$). **(B)** Tri/tetra-antennary branching ($\rho = -0.282$, $p = 0.308$). **(C)** Sialylation ($\rho = 0.150$, $p = 0.594$). **(D)** Core fucosylation ($\rho = 0.200$, $p = 0.475$). None supports a positive abundance association.
