# Supplementary material for: Glycan reachability analysis: A bottleneck-aware framework for inferring tissue-specific glycan biosynthetic potential from transcriptomics
Source: PLoS Comput Biol. 2026 Sep 24;22(9):e1014655. doi: 10.1371/journal.pcbi.1014655 (PMC13606954; doi:10.1371/journal.pcbi.1014655)
Supplement: Supplementary file 6 [file pcbi.1014655.s006.pdf]

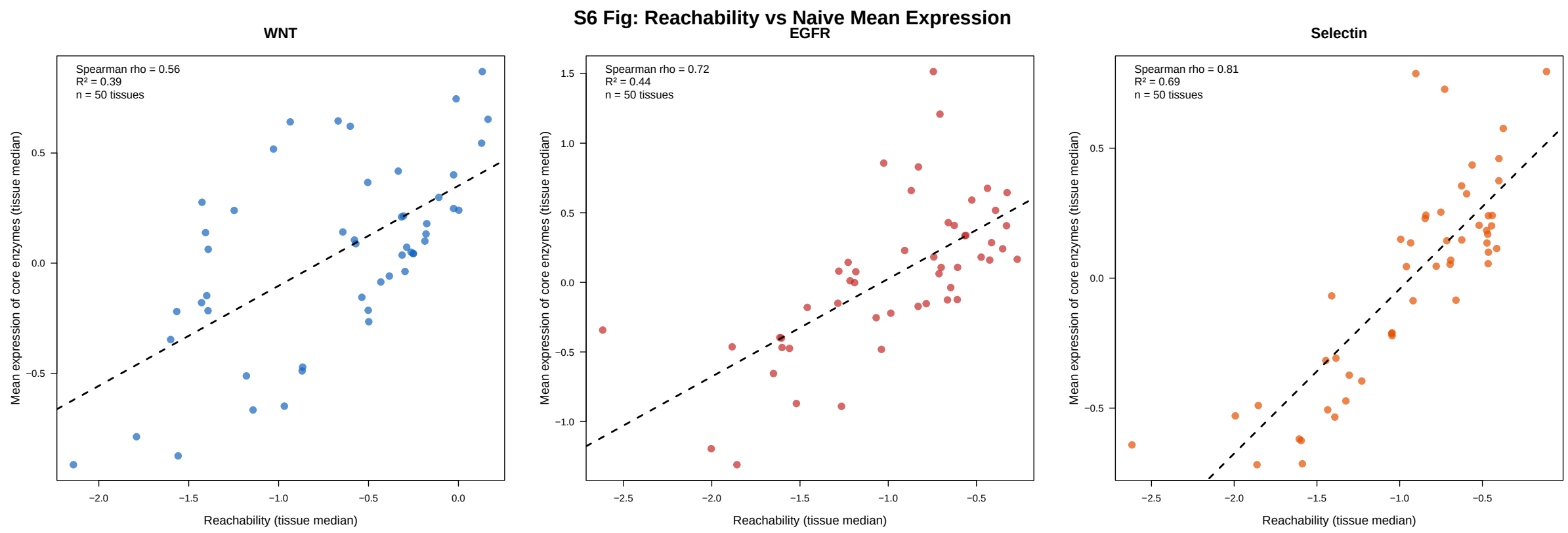

S6 Fig. Reachability versus naive mean expression. Scatter plots comparing tissue-median reachability and naive mean expression for the three coherence cascades. Divergence describes different aggregation behavior; it does not identify which score better reflects glycan abundance.
