# Supplementary material for: Glycan reachability analysis: A bottleneck-aware framework for inferring tissue-specific glycan biosynthetic potential from transcriptomics
Source: PLoS Comput Biol. 2026 Sep 24;22(9):e1014655. doi: 10.1371/journal.pcbi.1014655 (PMC13606954; doi:10.1371/journal.pcbi.1014655)
Supplement: Supplementary file 7 [file pcbi.1014655.s007.pdf]

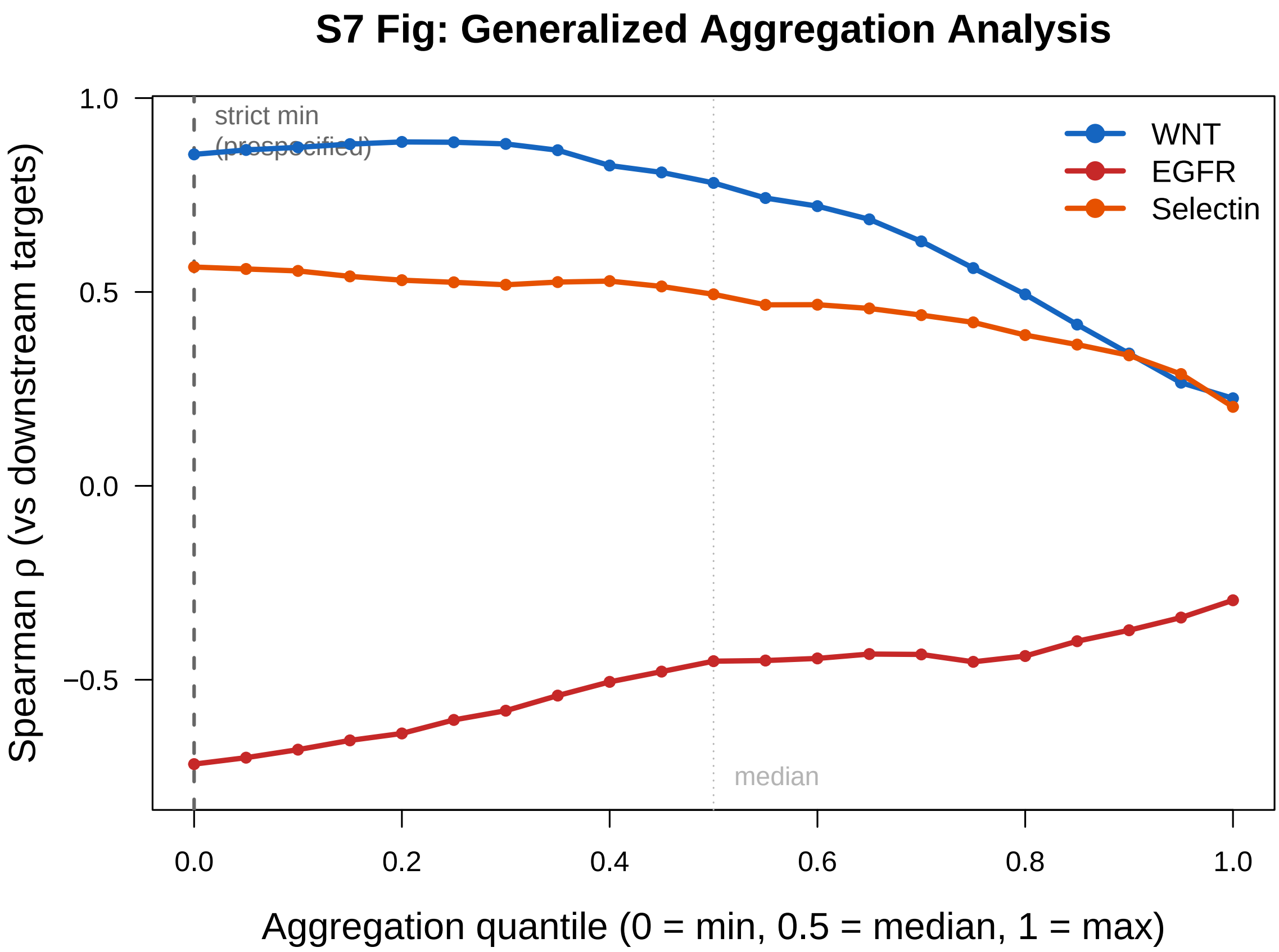

S7 Fig. AND-aggregation quantile sensitivity. Within-GTEx direction-oriented response correlation as the AND quantile varies from minimum ($q = 0$) to maximum ($q = 1$), with OR=mean fixed. Negative EGFR values are opposite the prespecified GM3-inhibitory direction. The sweep is descriptive and was not used to select the method.
