# Supplementary material for: Glycan reachability analysis: A bottleneck-aware framework for inferring tissue-specific glycan biosynthetic potential from transcriptomics
Source: PLoS Comput Biol. 2026 Sep 24;22(9):e1014655. doi: 10.1371/journal.pcbi.1014655 (PMC13606954; doi:10.1371/journal.pcbi.1014655)
Supplement: Supplementary file 8 [file pcbi.1014655.s008.pdf]

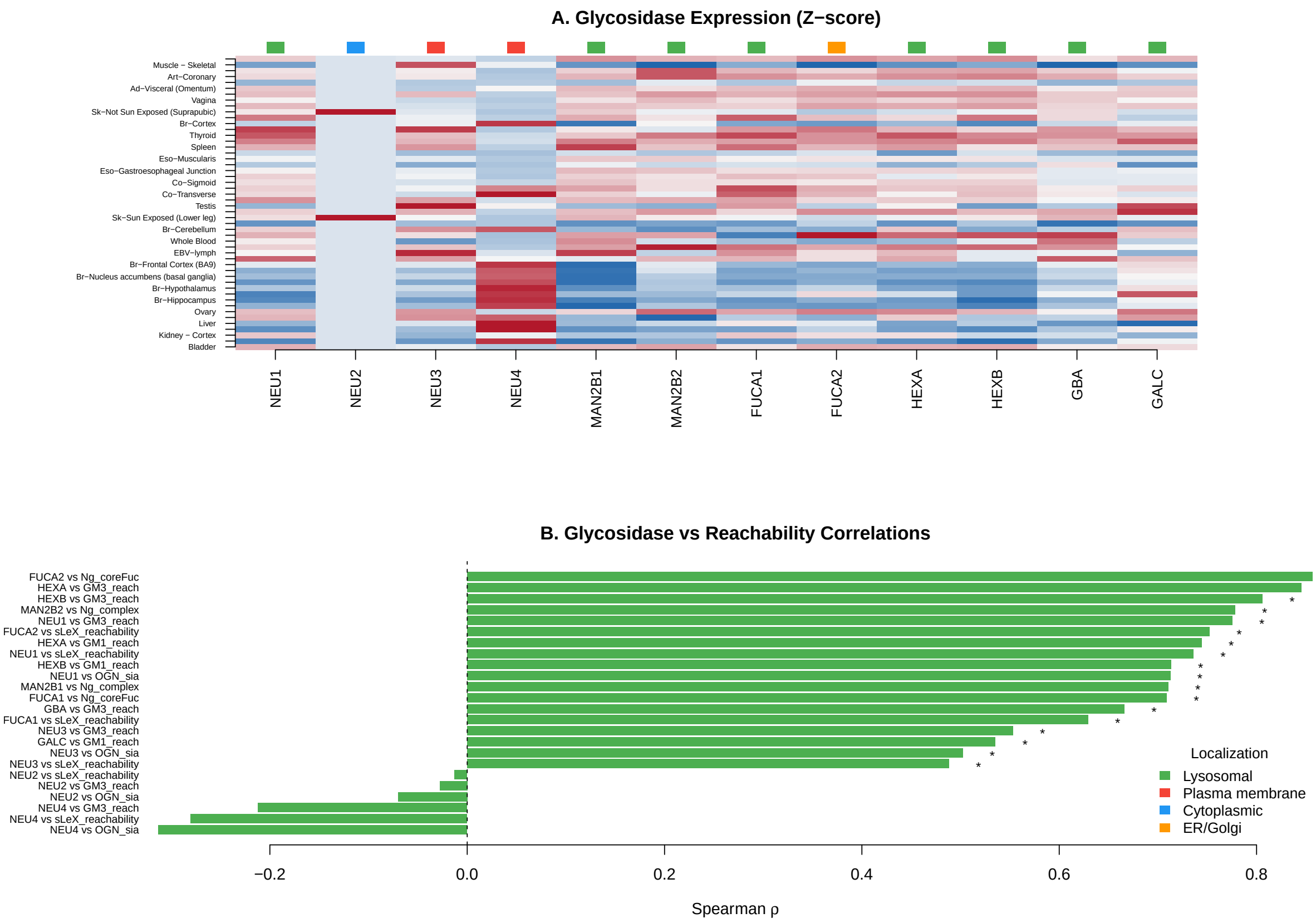

S8 Fig. Glycosidase expression survey. Spearman correlations between glycosidase expression and prespecified reachability metrics across 50 tissues. Examples are NEU1 versus GM3 ($\rho = 0.775$) and MAN2B1 versus Ng complex ($\rho = 0.711$). These shared-transcriptome associations neither measure turnover nor remove catabolic confounding.
