# Supplementary material for: Glycan reachability analysis: A bottleneck-aware framework for inferring tissue-specific glycan biosynthetic potential from transcriptomics
Source: PLoS Comput Biol. 2026 Sep 24;22(9):e1014655. doi: 10.1371/journal.pcbi.1014655 (PMC13606954; doi:10.1371/journal.pcbi.1014655)
Supplement: Supplementary file 9 [file pcbi.1014655.s009.pdf]

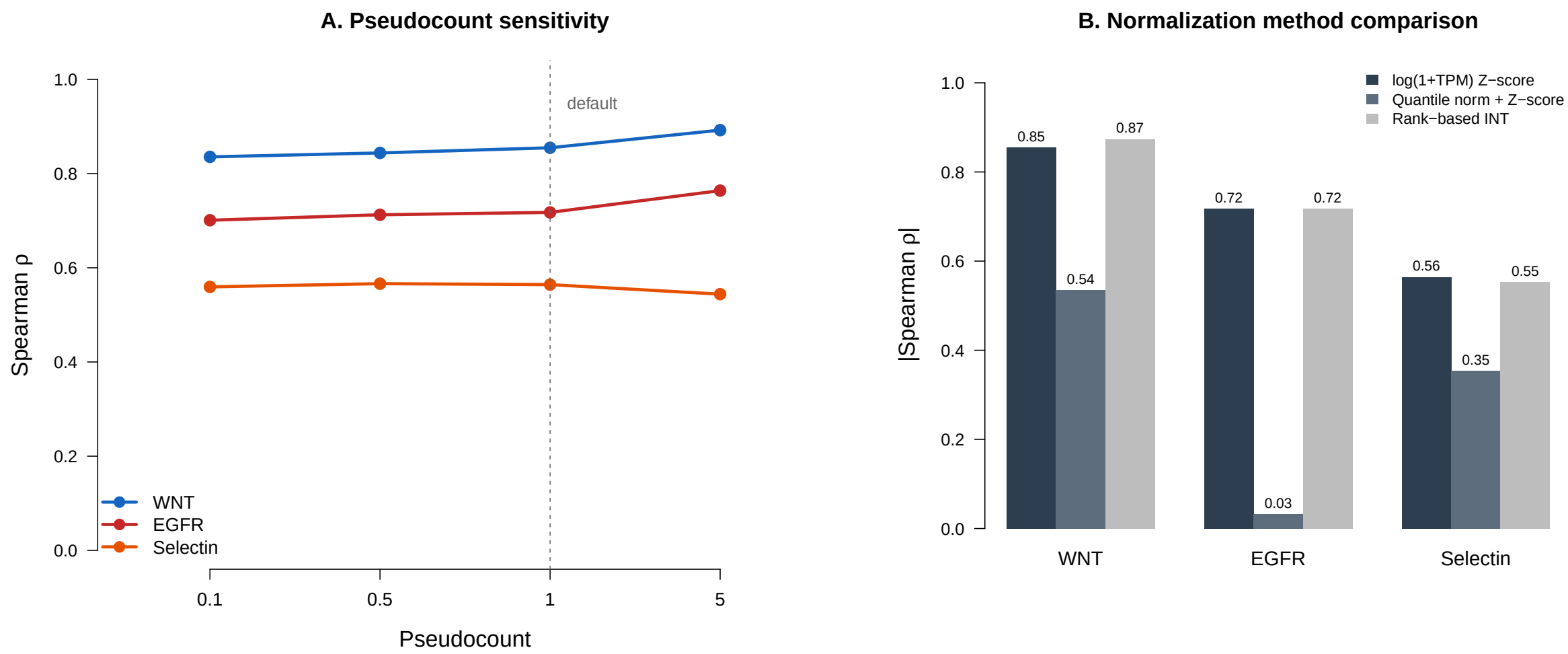

S9 Fig. Normalization and pseudocount sensitivity. Direction-oriented coherence correlations under four pseudocounts (0.1, 0.5, 1, 5), quantile normalization, and rank-based inverse-normal transformation. Negative EGFR values are opposite the prespecified GM3-inhibitory direction. Rank normalization is similar to the default, whereas quantile normalization substantially changes the correlations.
