# Supplementary material for: Glycan reachability analysis: A bottleneck-aware framework for inferring tissue-specific glycan biosynthetic potential from transcriptomics
Source: PLoS Comput Biol. 2026 Sep 24;22(9):e1014655. doi: 10.1371/journal.pcbi.1014655 (PMC13606954; doi:10.1371/journal.pcbi.1014655)
Supplement: Supplementary file 10 [file pcbi.1014655.s010.pdf]

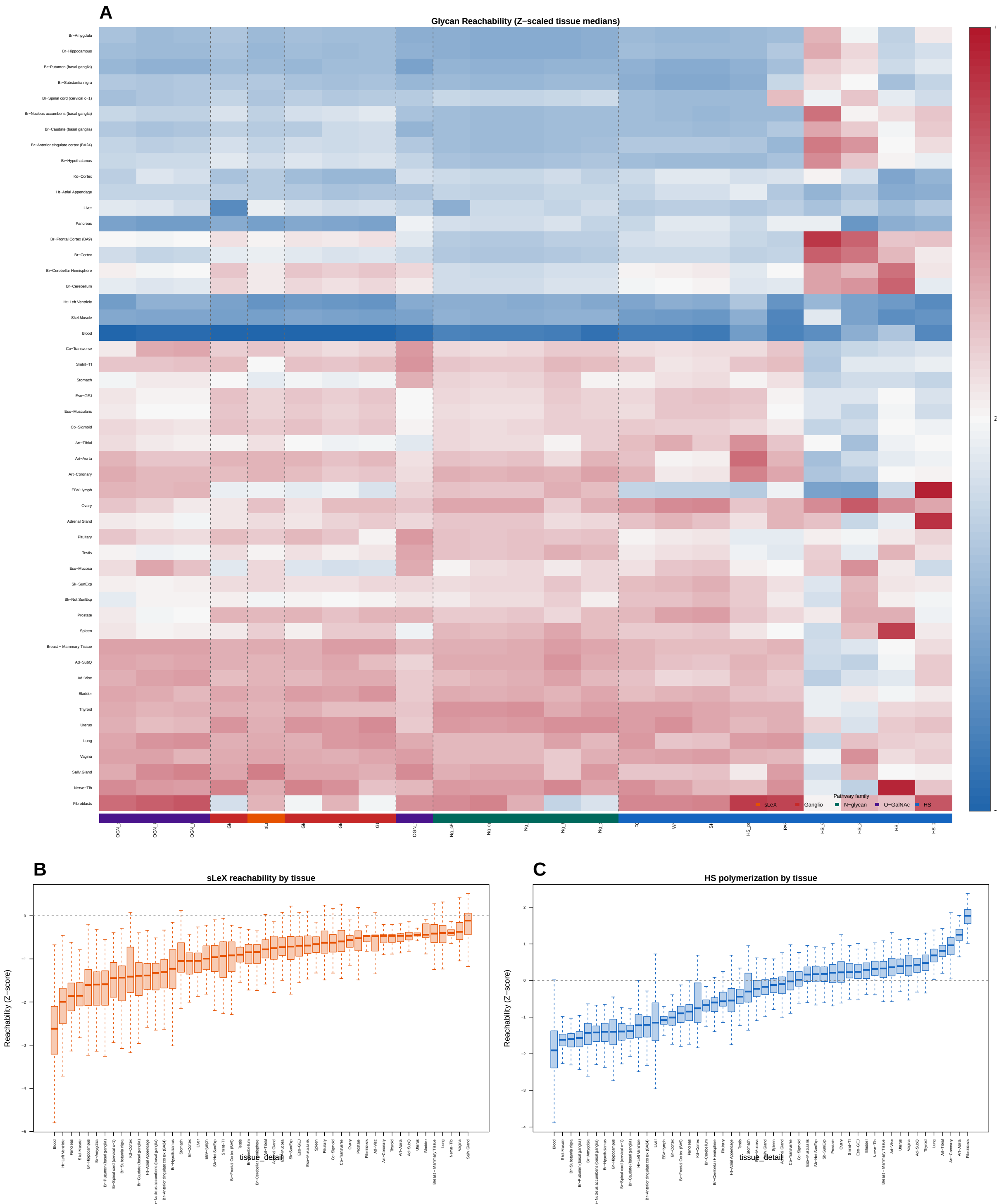

S10 Fig. Tissue median reachability landscape. Heatmap of Z-scaled tissue median reachability scores for all 23 metrics (rows) across 50 GTEx tissues (columns). Both axes are hierarchically clustered; tissue axis orientation and abbreviated labels match S1 Fig A for direct comparison. Brain regions form a tight cluster with elevated ganglioside (GM3, GD3) but depressed N-glycan and HS reachability. Connective tissue-rich organs (nerve, fibroblasts, ovary) show the highest HS-related reachability. Whole blood, skeletal muscle, and heart rank lowest across most metrics, reflecting reduced glycosylation machinery in terminally differentiated cells. This heatmap complements the raw expression landscape (S1 Fig A; S2 Fig) by revealing which biosynthetic pathways have expression-complete profiles rather than which individual genes are transcribed.
