# Supplementary material for: Glycan reachability analysis: A bottleneck-aware framework for inferring tissue-specific glycan biosynthetic potential from transcriptomics
Source: PLoS Comput Biol. 2026 Sep 24;22(9):e1014655. doi: 10.1371/journal.pcbi.1014655 (PMC13606954; doi:10.1371/journal.pcbi.1014655)
Supplement: Supplementary file 11 [file pcbi.1014655.s011.pdf]

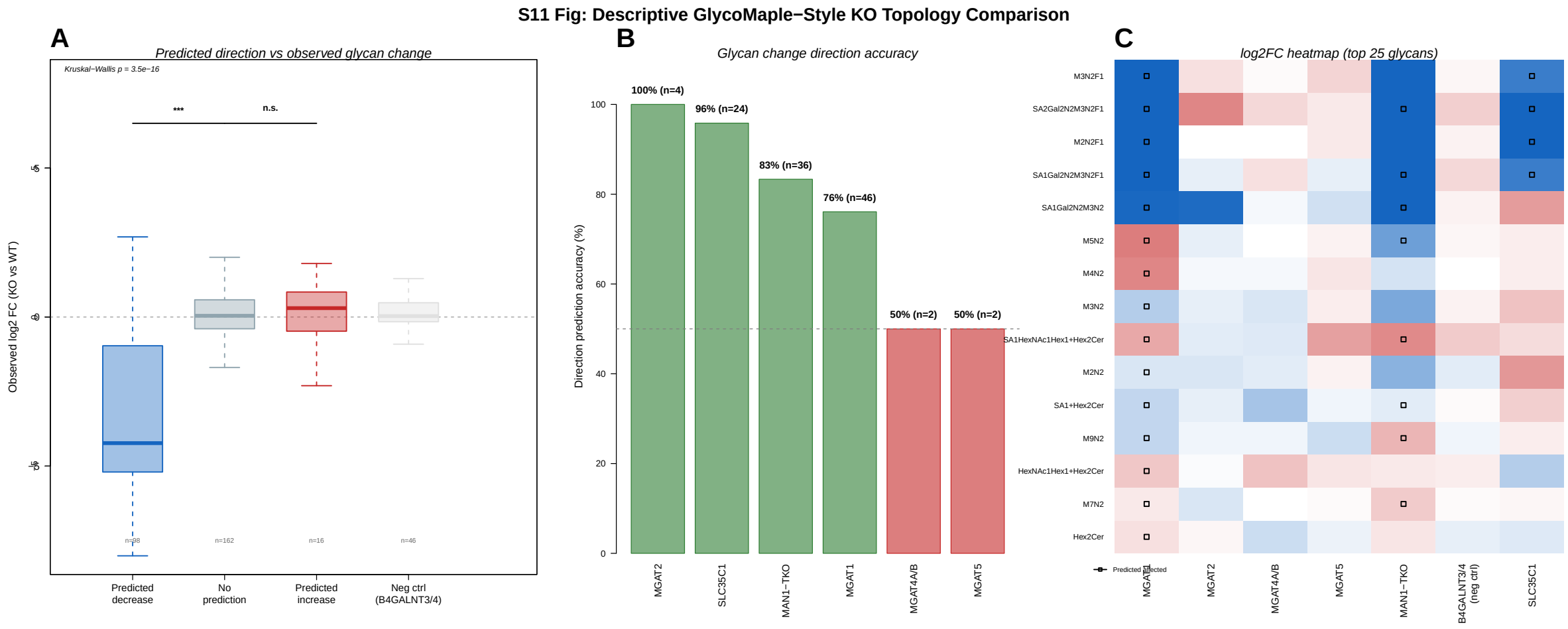

S11 Fig. Descriptive per-knockout topology detail. (**A**) Direction-prediction accuracy for six N-glycan-processing knockouts using HEK293 N-glycan mass-spectrometry data [1]. Bar height indicates the percentage of glycan species with correctly predicted direction of change. Early-pathway knockouts (SLC35C1, MGAT2, MGAT1, MAN1A1/A2/B1 triple) show high accuracy; late-pathway knockouts (MGAT4A/B, MGAT5) have few testable glycans. (**B**) Step expression rank versus glycomic perturbation magnitude. These panels are descriptive; the baseline-controlled benchmark is Fig 7, where the minimum does not outperform the mean or binary scorer.
