# Supplementary material for: Glycan reachability analysis: A bottleneck-aware framework for inferring tissue-specific glycan biosynthetic potential from transcriptomics
Source: PLoS Comput Biol. 2026 Sep 24;22(9):e1014655. doi: 10.1371/journal.pcbi.1014655 (PMC13606954; doi:10.1371/journal.pcbi.1014655)
Supplement: Supplementary file 12 [file pcbi.1014655.s012.pdf]

S1 Table. Donor substrate demand indices. Number of reachability metrics requiring each nucleotide sugar donor. CMP-Sia and UDP-Gal are the most widely shared donors (12 and 10 metrics, respectively). Metrics requiring multiple donors (e.g., sLeX requires CMP-Sia, GDP-Fuc, and UDP-Gal) have additional bottleneck opportunities.

Donor	#Metrics	Example metrics
CMP-Sia	12	sLeX, GM3, GD3, Ng_sia, OGN_sia
UDP-Gal	10	sLeX, GM1, Ng_complex, Ng_branch, OGN_Core1
GDP-Fuc	4	sLeX, Ng_coreFuc, reach_FGF/WNT/SHH
PAPS	8	HS_N, HS_2O, HS_6O, HS_3O, reach_FGF/WNT/SHH
UDP-GalNAc	6	GM2, OGN_Tn, OGN_Core1, OGN_Core2, OGN_sia
