# Supplementary material for: Glycan reachability analysis: A bottleneck-aware framework for inferring tissue-specific glycan biosynthetic potential from transcriptomics
Source: PLoS Comput Biol. 2026 Sep 24;22(9):e1014655. doi: 10.1371/journal.pcbi.1014655 (PMC13606954; doi:10.1371/journal.pcbi.1014655)
Supplement: Supplementary file 13 [file pcbi.1014655.s013.pdf]

S2 Table. Summary of within-GTEx coherence sensitivities. These statistics assess RNA-level association robustness, not glycan-mediated causation. Values are direction-oriented: the EGFR response was multiplied by -1 so that positive would match the prespecified GM3-inhibitory direction; negative EGFR values therefore point in the opposite direction. Complete outputs are in Supplementary File S2.

Analysis	WNT	EGFR	Selectin	Concern addressed
Primary ρ	0.855	-0.718	0.564	Tissue-level coherence
Donor-bootstrap 95% CI	[0.852, 0.873]	[-0.742, -0.700]	[0.536, 0.581]	Shared donors
Variance-matched perm. p	0.002	0.395	0.783	Generic tissue covariance
Within-tissue median ρ	0.549	-0.385	0.354	Tissue identity
Partial ρ (core mean)	0.795	-0.743	0.508	Conditional association
Partial ρ (all-pathway mean)	0.680	-0.641	0.387	Stricter expression control
