# Supplementary material for: Glycan reachability analysis: A bottleneck-aware framework for inferring tissue-specific glycan biosynthetic potential from transcriptomics
Source: PLoS Comput Biol. 2026 Sep 24;22(9):e1014655. doi: 10.1371/journal.pcbi.1014655 (PMC13606954; doi:10.1371/journal.pcbi.1014655)
Supplement: Supplementary file 14 [file pcbi.1014655.s014.pdf]

S3 Table. GTEx v8 tissue sample sizes. The table lists all 54 tissue types. Primary analyses were restricted to the 50 tissues with $N \geq 20$; Cervix Ectocervix, Cervix Endocervix, Fallopian Tube, and Kidney Medulla were excluded.

Tissue	N	Tissue	N
Adipose - Subcutaneous	663	Liver	226
Adipose - Visceral	541	Lung	578
Adrenal Gland	258	Minor Salivary Gland	162
Artery - Aorta	432	Muscle - Skeletal	803
Artery - Coronary	240	Nerve - Tibial	619
Artery - Tibial	663	Ovary	180
Bladder	21	Pancreas	328
Brain - Amygdala	152	Pituitary	283
Brain - Ant. cing. cortex	176	Prostate	245
Brain - Caudate	246	Skin - Not Sun Exp	604
Brain - Cb. Hemisphere	215	Skin - Sun Exposed	701
Brain - Cerebellum	241	Small Intestine	187
Brain - Cortex	255	Spleen	241
Brain - Frontal Cortex	209	Stomach	359
Brain - Hippocampus	197	Testis	361
Brain - Hypothalamus	202	Thyroid	653
Brain - Nuc. accumbens	246	Uterus	142
Brain - Putamen	205	Vagina	156
Brain - Spinal cord	159	Whole Blood	755
Brain - Subst. nigra	139		
Breast	459		
Fibroblasts	504		
Cervix - Ectocervix	9		
Cervix - Endocervix	10		
Colon - Sigmoid	373		
Colon - Transverse	406		
EBV-lymphocytes	174		
Esophagus - GEJ	375		
Esophagus - Mucosa	555		
Esophagus - Muscularis	515		
Fallopian Tube	9		
Heart - Atrial App.	429		
Heart - Left Ventricle	432		
Kidney - Cortex	85		
Kidney - Medulla	4		

S1 File. Machine-readable pathway definitions. Complete definitions of the five pathway families, 23 metrics, donor-supply chains, and OR=mean/AND=min rules in XLSX format.

S2 File. Complete benchmark and sensitivity analyses. Workbook containing KO benchmark pairs and summaries, nested quantile selection, all 950 tissue–metric bottleneck-stability rows, ST6GAL sensitivity results, and multiple-testing families.

References

[1] Huang YF, Aoki K, Akase S, Ishihara M, Liu YS, Yang G, et al. Global mapping of glycosylation pathways in human-derived cells. *Dev Cell.* 2021;56(8):1195–1209.

[2] Otaki M, Hirane N, Natsume-Kitatani Y, Nogami Itoh M, Shindo M, Kurebayashi Y, et al.
