# Supplementary material for: Glycan reachability analysis: A bottleneck-aware framework for inferring tissue-specific glycan biosynthetic potential from transcriptomics
Source: PLoS Comput Biol. 2026 Sep 24;22(9):e1014655. doi: 10.1371/journal.pcbi.1014655 (PMC13606954; doi:10.1371/journal.pcbi.1014655)
Supplement: Supplementary file 15 [file pcbi.1014655.s015.pdf]

S4 Table. Master list of all 23 reachability metrics. Each metric represents a distinct endpoint or pathway module. All 23 metrics are included in the pairwise tissue analysis; the Type column preserves the original endpoint/composite classification. Code variables match the analysis outputs.

Code variable	Display name	Pathway family	Type	#Steps	#Genes
sLeX_reachability	sLeX reachability	Sialyl Lewis X	Primary	3	12
GM3_reach	GM3 reach	Ganglioside	Primary	3	4
GM2_reach	GM2 reach	Ganglioside	Primary	4	5
GM1_reach	GM1 reach	Ganglioside	Primary	5	6
GD3_reach	GD3 reach	Ganglioside	Primary	4	5
HS_poly	HS polymerization	Heparan sulfate	Primary	1	2
PAPS	PAPS	Donor supply	Primary	2	4
HS_N	HS N-sulfation	Heparan sulfate	Primary	2	6
HS_2O	HS 2-O sulfation	Heparan sulfate	Primary	2	3
HS_6O	HS 6-O sulfation	Heparan sulfate	Primary	2	5
HS_3O	HS 3-O sulfation	Heparan sulfate	Primary	2	9
reach_FGF_like	HS FGF-like	HS composite	Composite	4	11
reach_WNT_like	HS WNT-like	HS composite	Composite	4	11
reach_SHH_like	HS SHH-like	HS composite	Composite	5	20
Ng_complex	Ng complex	N-glycan	Composite	7	21
Ng_branch	Ng branch	N-glycan	Composite	8	24
Ng_coreFuc	Ng core Fuc	N-glycan	Composite	8	22
Ng_bisect	Ng bisect	N-glycan	Composite	8	22
Ng_sia	Ng sialylation	N-glycan	Composite	8	23
OGN_Tn	OGN Tn	O-GalNAc	Composite	1	20
OGN_Core1	OGN Core 1	O-GalNAc	Composite	2	22
OGN_Core2	OGN Core 2	O-GalNAc	Composite	3	23
OGN_sia	OGN sialylation	O-GalNAc	Composite	3	24
