# Supplementary material for: Glycan reachability analysis: A bottleneck-aware framework for inferring tissue-specific glycan biosynthetic potential from transcriptomics
Source: PLoS Comput Biol. 2026 Sep 24;22(9):e1014655. doi: 10.1371/journal.pcbi.1014655 (PMC13606954; doi:10.1371/journal.pcbi.1014655)
Supplement: Supplementary file 16 [file pcbi.1014655.s016.pdf]

S5 Table. Tissue name abbreviations used in figures. Mapping between full GTEx v8 tissue names and abbreviated labels used in heatmaps and scatter plots throughout this study. Tissues whose full name is used without abbreviation (e.g., Liver, Lung, Ovary) are omitted. N: number of RNA-seq samples per tissue.

GTEx tissue name	Abbreviation	N
Adipose - Subcutaneous	Ad-SubQ	663
Adipose - Visceral (Omentum)	Ad-Visc	541
Adrenal Gland	Adrenal	258
Artery - Aorta	Art-Aorta	432
Artery - Coronary	Art-Coronary	240
Artery - Tibial	Art-Tibial	663
Brain - Amygdala	Br-Amygdala	152
Brain - Anterior cingulate cortex (BA24)	Br-ACC	176
Brain - Caudate (basal ganglia)	Br-Caudate	246
Brain - Cerebellar Hemisphere	Br-CbHemi	215
Brain - Cerebellum	Br-Cerebellum	241
Brain - Cortex	Br-Cortex	255
Brain - Frontal Cortex (BA9)	Br-FrCortex	209
Brain - Hippocampus	Br-Hippocampus	197
Brain - Hypothalamus	Br-Hypothalamus	202
Brain - Nucleus accumbens (basal ganglia)	Br-NAcc	246
Brain - Putamen (basal ganglia)	Br-Putamen	205
Brain - Spinal cord (cervical c-1)	Br-SpCord	159
Brain - Substantia nigra	Br-SN	139
Breast - Mammary Tissue	Breast	459
Cells - Cultured fibroblasts	Fibroblasts	504
Cells - EBV-transformed lymphocytes	EBV-lymph	174
Colon - Sigmoid	Co-Sigmoid	373
Colon - Transverse	Co-Transverse	406
Esophagus - Gastroesophageal Junction	Eso-GEJ	375
Esophagus - Mucosa	Eso-Mucosa	555
Esophagus - Muscularis	Eso-Muscularis	515
Heart - Atrial Appendage	Ht-Atrial Appendage	429
Heart - Left Ventricle	Ht-Left Ventricle	432
Kidney - Cortex	Kd-Cortex	85
Kidney - Medulla	Kd-Medulla	4
Minor Salivary Gland	Saliv.Gland	162
Muscle - Skeletal	Skel.Muscle	803
Nerve - Tibial	Nerve-Tib	619
Skin - Not Sun Exposed (Suprapubic)	Sk-NoSun	604
Skin - Sun Exposed (Lower leg)	Sk-SunExp	701
Small Intestine - Terminal Ileum	SmInt-TI	187
Whole Blood	Blood	755
