# Supplementary material for: Glycan reachability analysis: A bottleneck-aware framework for inferring tissue-specific glycan biosynthetic potential from transcriptomics
Source: PLoS Comput Biol. 2026 Sep 24;22(9):e1014655. doi: 10.1371/journal.pcbi.1014655 (PMC13606954; doi:10.1371/journal.pcbi.1014655)
Supplement: Supplementary file 17 [file pcbi.1014655.s019.pdf]

Manuscript Number: PCOMPBIOL-D-26-00715

Glycan Reachability Analysis: A Bottleneck-Aware Framework for Inferring Tissue-Specific Glycan Biosynthetic Potential from Transcriptomics

Manuscript Type: Methods

The present manuscript introduces a glycan biosynthetic reachability framework that uses transcriptomic pathway logic to infer tissue-specific glycan biosynthetic potential from GTEx RNA-seq data. The work is interesting, timely, and useful for computational glycobiology. The central idea, using AND/OR pathway logic and a bottleneck-aware aggregation strategy to move beyond binary enzyme detectability, is a clear conceptual contribution. The manuscript is also transparent about limitations, particularly the distinction between transcriptomic potential and actual glycan abundance or enzymatic activity. Prior to publication, the author is requested to address the following comments:

1. The manuscript could provide a broader representation of existing literature on glycan biosynthesis modelling, which would strengthen its scope and contributions to the field.

2. Because both reachability scores and downstream target scores are derived from the same GTEx RNA-seq data, the analysis does not demonstrate that glycan products mediate the observed associations. It demonstrates transcriptomic coherence between glycosylation-related genes and selected pathway-response genes. This is still valuable, but the language should be calibrated accordingly. Ideally, the authors should include an orthogonal validation layer, such as matched glycomics, glycoproteomics, proteomics, or perturbation data. If this is not feasible, the manuscript should use more precise language that reflects such correlations.

3. The bottleneck principle is the central methodological choice. It is useful and interpretable, but a strict minimum can be driven by a single noisy, lowly expressed, or poorly annotated gene. I would encourage the author to report bottleneck stability and/or show whether the same limiting step is repeatedly identified within tissues. This would make the "bottleneck" interpretation more robust.

4. The failed cross-species glycomics comparison and largely negative DepMap essentiality analysis are reported transparently, which is commendable. However, the negative glycomics comparison is especially relevant because direct glycan abundance prediction is the most biologically meaningful external validation. The authors should discuss more explicitly how this affects confidence in the reachability score as a proxy for glycan biosynthetic potential.
