# Supplementary material for: Glycan reachability analysis: A bottleneck-aware framework for inferring tissue-specific glycan biosynthetic potential from transcriptomics
Source: PLoS Comput Biol. 2026 Sep 24;22(9):e1014655. doi: 10.1371/journal.pcbi.1014655 (PMC13606954; doi:10.1371/journal.pcbi.1014655)
Supplement: Supplementary file 18 [file pcbi.1014655.s021.pdf]

Response to Reviewers - PCOMPBIOL-D-26-00715

Yusuke Matsui

I thank the editor and both reviewers for their careful and constructive assessment. The central concern was that the original within-transcriptome evaluation could not serve as a benchmark of biological accuracy. I have addressed this by adding an independent HEK293 knockout glycomics benchmark and by revising the paper's interpretation to match its result: reachability contains non-random topology-related information, but the strict minimum does not outperform naive mean aggregation or binary topology. The former signaling analysis is now presented only as within-GTEx transcriptomic coherence, and the negative tissue-glycome evidence is given greater weight. The responses below explain how each comment changed the analysis, interpretation, and manuscript.

Navigation note. Each item is organized as requested: reviewer comment, author response, then a Location/Revision summary stating where and how the manuscript was changed, followed by selected exact revised text. The Location field lists every substantive main-manuscript and Supporting Information change attributable to that comment; important cross-cutting locations are repeated where they answer more than one comment. Exact quotations are limited to the passages most useful for review and are shown in blue. Line numbers refer to the clean revised main-manuscript PDF; its Supplementary Methods begin at line 524. Supporting Information figures and tables are cited by page because they are LaTeX floats. Substantive corrections not attributable to one reviewer comment are listed separately at the end. Pure typesetting, pagination, and metadata changes are not listed. The three figures most directly bearing on the editorial decision are reproduced once: new Fig 7, revised Fig 2A-B, and S5 Fig.

Editor: benchmarking

Editor comment. *Thank you for you manuscript on the analysis of glycosylation related transcriptomics. Given the points made by Reviewer 1, particularly regarding the flawed evaluation method, I hesitated between rejection and major-revision. Given the novelty in the approach I am giving you a chance to address those critiques. However, I emphasize that after the next revision I will make an assessment of whether the issue of benchmarking has been addressed appropriately, if not I will have to reject the manuscript. As such, please make sure you carefully address the issue of benchmarking. Obviously, you should also address all other comments from both reviewers.*

Author response. I agree that the original evaluation was not a benchmark in the methodological sense because both the score and its proposed readouts came from the same transcriptomes. I therefore replaced that evidentiary role with an independent HEK293 knockout N-glycomics benchmark. It compares the strict minimum directly with two prespecified alternatives: naive mean aggregation and GlycoMaple-style binary topology. The important result is not a claim of victory for the proposed method. The minimum did not significantly outperform either comparator, although its pooled discrimination exceeded a label-permutation null. A nested leave-one-knockout-out analysis also selected a relaxed low quantile rather than the strict minimum. I have made these negative comparative results central to the revision and no longer present the strict minimum as validated or superior. This directly addresses the benchmarking concern while setting a narrower, evidence-supported role for reachability as a transcriptomic prioritization score.

Changes in the manuscript. Location: Main manuscript, Author Summary, p. 1; Abstract, lines 9-26; Methods Section 2.7, lines 133-193; Results Sections 3.7-3.10, lines 340-412; Discussion Sections 4.1-4.4, lines 414-521; Figs 5-7 and captions, pp. 31-33; Supplementary Methods, lines 532-642; Supporting Information, S3, S7, S9, and S11 Fig captions, pp. 4, 8, 11, and 13, and S2 Table, p. 14; S2 File. **Revision:** I replaced the original same-transcriptome evidentiary framework with an independent HEK293 knockout N-glycomics benchmark, prespecified minimum, mean, and binary comparators, and nested leave-one-knockout-out quantile selection. The Summary, Abstract, Results, Discussion, main figures, supplementary analyses, and captions now consistently report that the minimum was not superior, that $q = 0.2$ was preferred, and that the within-GTEx analyses are coherence analyses rather than biological validation.

Revised text.

> "Mean AUROC across the six strata was 0.786 for the minimum, 0.768 for the mean, and 0.802 for binary topology; mean AUPRC was 0.585, 0.582, and 0.591, respectively. Paired AUROC differences for minimum minus mean (0.018, bootstrap 95% CI $[-0.003, 0.039]$, exact $p = 0.25$) and minimum minus binary (-0.016, $[-0.054, 0.017]$, exact $p = 0.625$) did not support superiority of the minimum." (lines 381-385)

Revised figure caption (new Fig 7).

> "External benchmark against knockout glycomics. Three prespecified, parameter-free scorers were evaluated across six HEK293 knockout strata; B4GALNT3/4 was an off-pathway negative control. (A) Mean $\pm$ s.d. AUROC across knockout strata. Minimum scoring did not outperform mean aggregation or binary topology. (B) Per-knockout AUROC, showing heterogeneity among perturbations. (C) For the only selected parameter, q, nested leave-one-knockout-out evaluation chose q on five knockouts and scored the sixth. Every fold selected $q = 0.2$ and outer-fold mean AUROC was 0.811, favoring a relaxed low quantile rather than validating the strict minimum." (Fig 7 caption, p. 33)

Revised supporting-figure caption (S11 Fig).

> "These panels are descriptive; the baseline-controlled benchmark is Fig 7, where the minimum does not outperform the mean or binary scorer." (S11 Fig caption, p. 13)

New Fig 7 reproduced for reviewer convenience.

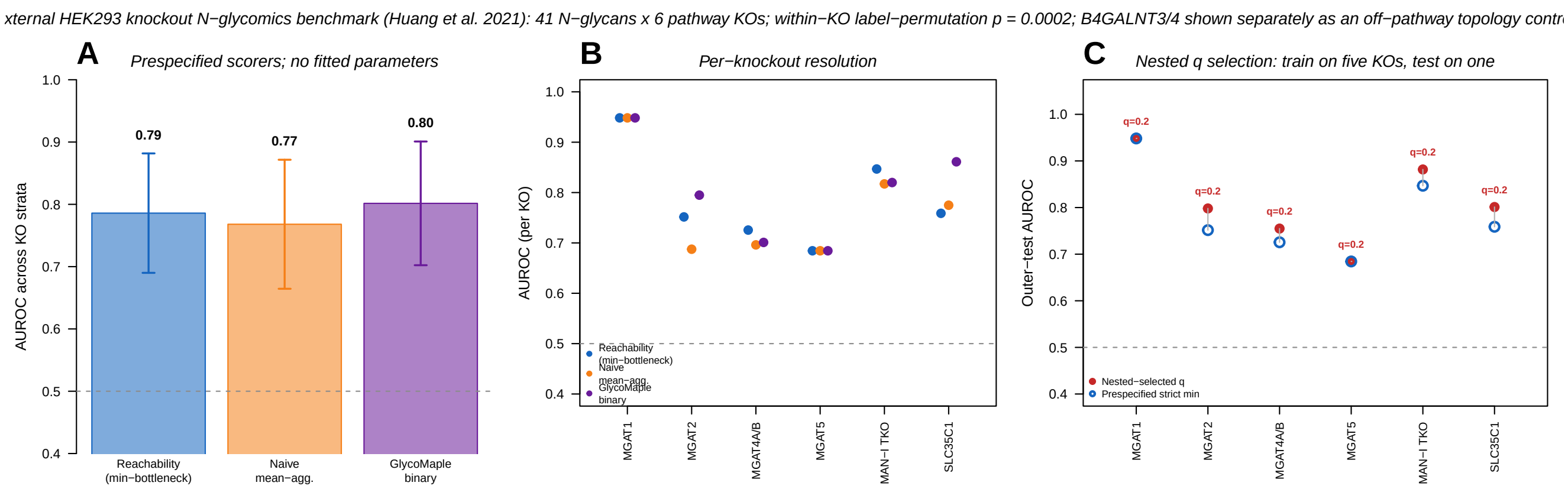

Reviewer 1

R1.1 - Relationship to glycan abundance and practical impact

Reviewer comment. *The author states "We emphasize that reachability scores measure transcriptomic biosynthetic potential, not enzymatic activity or glycan abundance", yet the entire impact of this article hinges on the relationship to glycan abundance. To illustrate this, the author then contradicts their own limitation later with approaches such as the downstream signaling evaluation, which is implicitly based on assumptions of the impact of the glycans themselves. It is not made evident that there would be genuine applications for "transcriptomic biosynthetic potential" that would not (explicitly or implicitly) be actually targeted to the produced glycan/glycan epitope. Unrealized potential, in other words, does not seem to be of practical interest.*

In fact, there are hints in the manuscript that reachability does not reflect glycosylation, such as the unpromising ganglioside reachability assigned to the brain, the region with the highest ganglioside in the body. Similarly, while the transparency is appreciated and acknowledged, the null-finding in the cross-species tissues comparison further indicates that reachability does not yield insights into the produced glycome.

Author response. The reviewer is correct that the original framing blurred transcriptomic potential with realized glycan abundance and therefore overstated practical impact. I have resolved that contradiction by narrowing the claim. Reachability is now presented as a hypothesis-generating rank for prioritizing tissue-pathway pairs and candidate expression-limiting steps for direct measurement, not as a substitute for glycomics. The brain-ganglioside discrepancy and the null mouse comparison are no longer treated as anomalies to explain away; they are explicit counter-evidence that lowers confidence in tissue-level abundance interpretation. The new knockout benchmark provides an orthogonal perturbation-glycomics test, but it does not rescue the broader abundance claim because it does not show superiority of the minimum and does not test steady-state tissue abundance. The revised manuscript therefore states both the limited practical use that remains and the direct measurements required before stronger biological claims can be made.

Changes in the manuscript. Location: Main manuscript, Author Summary, p. 1; Abstract, lines 9-26; Introduction, lines 52-68; Results Sections 3.2-3.4, lines 218-299, Section 3.7, lines 340-362, and Section 3.10, lines 394-412; Discussion Sections 4.2-4.4, lines 448-521; Fig 2 and caption, pp. 26-27, and Fig 5 and caption, p. 31; Supporting Information, S3-S6 and S8 Fig captions, pp. 4-7 and 10; S2 File. **Revision:** I narrowed the claimed use of reachability to hypothesis generation and

measurement prioritization throughout the Summary, Abstract, Introduction, Results, Discussion, and figure captions. I removed signaling and turnover interpretations as abundance evidence, elevated the brain discrepancy and null mouse tissue-glycome comparison as counter-evidence, treated DepMap as secondary, and specified the matched glycomics and perturbation data required for stronger claims.

Revised text.

> "We emphasize that reachability is a relative rank of transcriptomic biosynthetic potential, not a measure of enzymatic activity or glycan abundance. Its intended use is to prioritize tissue–pathway pairs and candidate limiting steps for direct glycomic measurement; whether the ranking reflects realized glycan abundance must be tested rather than assumed." (lines 62-66)

Revised figure caption (S5 Fig).

> "None supports a positive abundance association." (S5 Fig caption, p. 6)

R1.2 - Differences between methods are not evidence of superiority

Reviewer comment. *Related to this, differences of reachability vs other methods are portrayed as "interesting findings" but a mere difference (without method benchmarking etc), especially without founded claims of downstream impacts, carries little information that can be relied upon and should not be overinterpreted.*

Author response. I agree. A discordance between scores shows only that the representations behave differently; it does not establish which is biologically correct. I have removed the positive interpretation of the pancreas, nerve, and brain examples and now use them only to identify cases where matched glycomics would be informative. I also removed CA19-9 as indirect support for the normal-pancreas result because a malignant-tissue biomarker cannot adjudicate a normal-tissue prediction. Comparative accuracy is now addressed only by the independent knockout benchmark, which does not demonstrate superiority of the minimum. Thus, the revised manuscript separates descriptive resolution from validated accuracy rather than treating one as evidence for the other.

Changes in the manuscript. Location: Main manuscript, Author Summary, p. 1; Abstract, lines 9-26; Results Section 3.2, lines 218-237, Section 3.3, lines 238-275, and Sections 3.8-3.9, lines 363-393; Discussion Sections 4.1-4.2, lines 414-464; Fig 2 and caption, pp. 26-27, and Figs 6-7 and captions, pp. 32-33; Supporting Information, S6, S7, and S11 Fig captions, pp. 7, 8, and 13; S2 File. **Revision:** I removed the positive biological interpretation of score discordances and the CA19-9 argument, recast the pancreas, nerve, and brain examples as descriptive cases requiring matched glycomics, labeled the aggregation sweep as post-hoc sensitivity rather than evidence of accuracy, and restricted comparative claims to the independent benchmark, which did not show superiority of the minimum.

Revised text.

> "We present this discordance as an illustration of the resolution reachability adds over a binary call, not as evidence that reachability is correct here: from transcriptomics alone we cannot determine which representation better reflects the produced sLeX glycome, and matched normal-pancreas glycomics would be required to adjudicate." (lines 258-261)

Revised figure captions.

> "Discordance describes different score behavior and does not determine which better reflects glycan abundance." (Fig 2 caption, p. 26)

> "Divergence describes different aggregation behavior; it does not identify which score better reflects glycan abundance." (S6 Fig caption, p. 7)

Revised Fig 2A-B reproduced for reviewer convenience. These panels show the score discordances discussed above; they are not evidence that either representation better reflects glycan abundance.

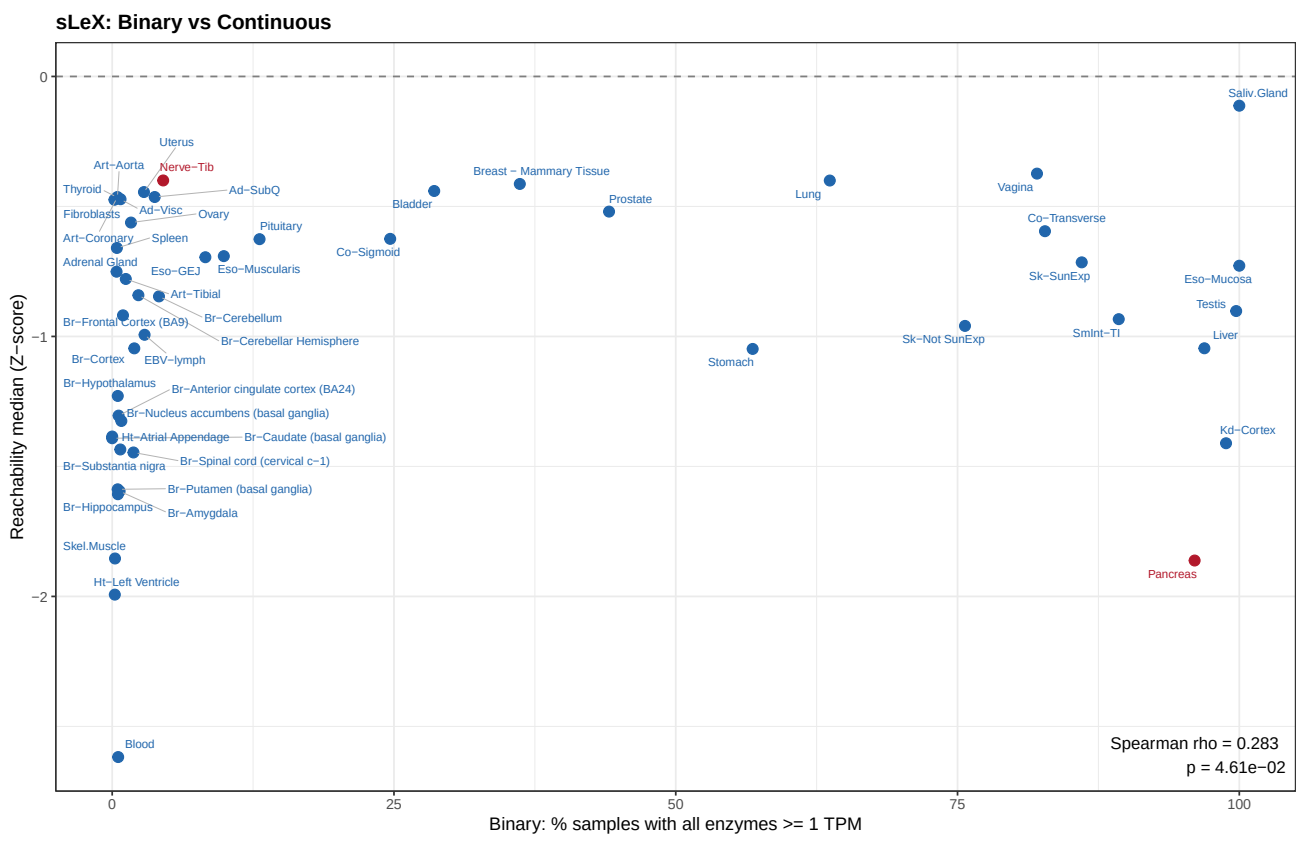

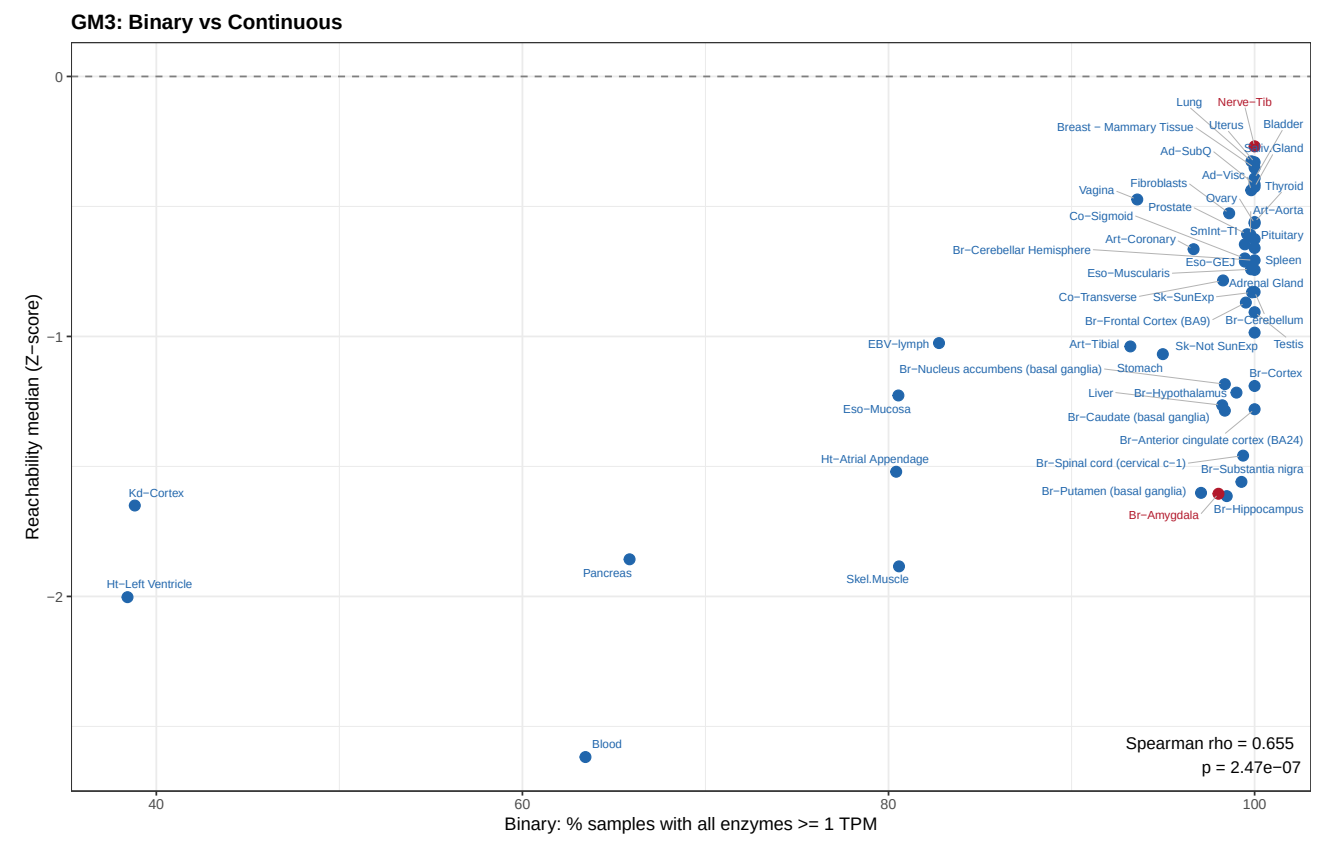

R1.3 - Indirect and confounded signaling analysis

Reviewer comment. *Validating the impact of reachability by looking at downstream signaling pathway outputs that are presumably affected by the change in glycosylation is an incredibly indirect and error/confounder-prone way of tackling this problem. Even if such a procedure would be validated/benchmarked (which it is not), it would be highly uncertain. It is also made conceptually impossible by the author's claim that reachability does not indicate produced glycans (which, as stated, are the supposed impact on signaling).*

Author response. I agree with the conceptual objection. Because both axes are computed from the same GTEx RNA-seq data, the analysis cannot validate glycan-mediated signaling. I have therefore removed the terms "functional validation" and "downstream consequence" and renamed the analysis transcriptomic coherence. The revised Results also disclose that the EGFR direction is opposite to the prespecified glycan-mediated hypothesis and that variance-matched random-gene-set testing does not retain the EGFR or Selectin associations. These findings are retained only to describe how the constructed score co-varies with transcriptional programs, not as evidence of mediation or biological accuracy. The independent knockout glycomics analysis in Fig 7 is now the only orthogonal benchmark.

Changes in the manuscript. Location: Main manuscript, Author Summary, p. 1; Abstract, lines 9-26; Methods Section 2.7, lines 133-193; Results Section 3.7, lines 340-362; Fig 5 and caption, p. 31; Discussion Section 4.3, lines 465-500; Supplementary Methods, lines 539-561, 597-629, and 636-642; Supporting Information, S3, S8, and S9 Fig captions, pp. 4, 10, and 11, and S2 Table, p. 14; S2 File. **Revision:** I renamed the analysis from functional validation/downstream consequence to transcriptomic coherence in every affected section and caption, stated that it cannot establish glycan-mediated signaling, reported the opposite EGFR direction and the failure of variance-matched random-gene-set tests to retain the EGFR or Selectin associations, removed the turnover interpretation of the glycosidase survey, and limited all associated robustness analyses to descriptive within-GTEx evidence.

Revised text.

> "We stress at the outset that this is a coherence analysis within a single RNA-seq dataset: both the reachability scores and the pathway-response readouts are derived from the same GTEx transcriptomes, so it cannot, and does not, establish that the glycan products mediate these associations — only that reachability co-varies across tissues with the relevant transcriptional programs." (lines 138-142)

Revised figure captions.

> "These are within-GTEx coherence analyses (predictors and readouts share an RNA-seq origin); they do not establish glycan-mediated causation (Section 3.7)." (Fig 5 caption, p. 31)

> "Both axes derive from the same RNA-seq and do not establish glycan-mediated signaling." (S3 Fig caption, p. 4)

R1.4 - Benchmark target, data splitting, and overfitting

Reviewer comment. *All benchmarking that is done in this article (e.g., different aggregation methods) is done with tasks that are themselves not validated, such as the signaling pathway analysis. Targets are being optimized for which the interpretation (and thus the impact/relevance) is very unclear. There is also no data splitting in this, so this essentially presents overfitting.*

Author response. I agree that using the same unvalidated within-GTEx task both to select and evaluate an aggregation rule would be circular and prone to overfitting. The within-GTEx aggregation sweep is therefore no longer used to choose or justify the method; it is labeled as post-hoc sensitivity analysis only. In the new external benchmark, the three principal

scorers are prespecified and have no fitted parameters. The only selected parameter, the aggregation quantile q, is evaluated by nested leave-one-knockout-out analysis, so each held-out knockout is scored once using a value selected without that knockout. The result selected $q = 0.2$ in every fold and therefore argues for a relaxed low quantile, not retrospective validation of the strict minimum.

Changes in the manuscript. Location: Main manuscript, Author Summary, p. 1; Abstract, lines 9-26; Methods Section 2.7, lines 133-193; Results Sections 3.7-3.9, lines 340-393; Figs 5-7 and captions, pp. 31-33; Supplementary Methods, lines 532-629; Supporting Information, S7, S9, and S11 Fig captions, pp. 8, 11, and 13, and S2 Table, p. 14; S2 File. **Revision:** I reclassified the within-GTEx aggregation sweep as post-hoc sensitivity rather than model selection, removed the same-RNA-seq task as a benchmark target, added three prespecified parameter-free scorers in the external benchmark, and evaluated the only selected parameter by nested leave-one-knockout-out analysis. The Summary, Abstract, figures, captions, and sensitivity results now state that the nested result favored $q = 0.2$ and does not validate the strict minimum.

Revised text.

> "Aggregation quantile was the only selected parameter and was assessed with nested leave-one-knockout-out evaluation: q was chosen on five knockouts and scored once on the sixth. Every outer fold selected $q = 0.2$, yielding mean outer-fold AUROC 0.811. This favors a relaxed low quantile rather than validating the strict minimum." (lines 389-392)

Revised figure captions.

> "These results compare internal RNA-level behavior and are not a glycan-accuracy benchmark." (Fig 6 caption, p. 32)
>
> "The sweep is descriptive and was not used to select the method." (S7 Fig caption, p. 8)

R1.5 - Novelty of the tissue-expression survey

Reviewer comment. *Especially for the early results section, it is unclear whether the mere analysis of glycosyltransferase expression across tissues is novel (at the very least it should be placed into context with the many studies that did exactly that, and what they have found).*

Author response. I agree that the descriptive expression landscape is established context rather than a novel result. I have added prior tissue and single-cell glycogene-expression studies and now use the survey only to show that GTEx recapitulates known tissue organization before quantitative pathway integration is introduced. The manuscript's novelty claim is restricted to the bottleneck-aware continuous integration and its reliability diagnostics, not the gene-expression survey itself.

Changes in the manuscript. Location: Main manuscript, Results Section 3.1, lines 194-206, including the added Nairn et al. tissue-expression citation in the References. **Revision:** I added prior tissue and single-cell glycogene-expression studies, recast the tissue-expression survey as confirmation that GTEx reproduces established tissue organization, and restricted the novelty claim to bottleneck-aware continuous pathway integration and its reliability diagnostics.

Revised text.

> "We therefore present this landscape not as a novel finding but as the premise for our method — confirming that GTEx recapitulates the known organization before asking whether quantitative pathway integration adds resolution beyond a gene-by-gene view. The contribution of this study is not the expression landscape but the bottleneck-aware, continuous reachability integration that follows." (lines 203-207)

R1.6 - Compensation, moonlighting, uncertainty, and FUT8

Reviewer comment. *A more general point that the author should at least discuss. Some clear steps, such as core fucosylation & FUT8, demonstrate the potential pitfalls might encounter. For, if FUT8 is knocked out, such as here (https://www.ahajournals.org/doi/10.1161/ATVBAHA.126.324757), core fucosylation can still be detected, just at lower levels. So, ideally, modeling strategies would also model an uncertainty/error term, for unknown enzyme moonlighting activities/compensation, etc.*

Author response. I agree that a deterministic minimum cannot represent unknown compensation, enzyme promiscuity, annotation error, or post-transcriptional regulation. The reviewer-provided FUT8 example is now cited because it directly demonstrates why setting the canonical enzyme to zero does not guarantee a zero product signal. I have therefore changed the interpretation of the argmin from a biochemical rate-limiting step to an expression-limiting position. I did not add an unparameterized error term, which would create an appearance of uncertainty quantification without an empirical basis. Instead, the limitation is stated explicitly and a probabilistic per-step model, calibrated against perturbation data, is identified as the appropriate future extension.

Changes in the manuscript. Location: Main manuscript, Abstract, lines 9-26; Methods Section 2.2, lines 84-101; Results Section 3.2, lines 218-237, and Section 3.4, lines 276-299; Discussion Section 4.1, lines 414-447, and Section 4.3, lines 465-500; Fig 1 caption, p. 25, and Fig 2D caption, p. 27; Supplementary Methods, lines 562-572 and 615-617; Supporting Information, S7 Fig caption, p. 8; S2 File; the reviewer-provided FUT8 study in the References. **Revision:** I cited the FUT8 example, replaced biochemical rate-limiting language with expression-limiting position/argmin throughout the affected text and captions, added donor-level stability diagnostics, explicitly listed compensation, promiscuity, annotation error, and post-transcriptional regulation as unmodeled uncertainties, reported deterministic aggregation sensitivity, and identified a perturbation-calibrated probabilistic model as the appropriate future extension.

Revised text.

> "The deterministic minimum also lacks an uncertainty term for annotation error, redundancy, compensation, or enzyme promiscuity." (lines 481-482)

> "A probabilistic per-step extension that propagates expression and annotation uncertainty is therefore a priority for future work." (lines 490-491)

Revised figure caption (Fig 1).

> "The score is the minimum across required positions; the argmin is an expression-limiting position, not a proven biochemical rate-limiting step." (Fig 1 caption, p. 25)

R1.7 - ST6GAL1/2 averaging

Reviewer comment. *Given that reachability scores depend on isozyme means, ST6GAL1/2 may not be the best choice, since ST6GAL2 is barely expressed and will always artificially drag down the workhorse ST6GAL1.*

Author response. The concern is well founded: the prespecified OR=mean rule can dilute ST6GAL1 when ST6GAL2 is weakly expressed. I tested the consequence directly by recomputing the relevant metric with OR=max and with ST6GAL1 alone. Overall tissue rankings remained highly concordant, although individual tissues changed materially. Thus, the dilution does not drive the global ranking, but it is a real source of local sensitivity and is now reported as such. I retained the submitted OR=mean rule to avoid redefining the method after seeing the review result, while providing the alternative analyses needed to assess this specific concern.

Changes in the manuscript. Location: Main manuscript, Methods Sections 2.1-2.2, lines 71-101; Results Section 3.2, lines 218-237, and Section 3.8, lines 363-371; Fig 6 and caption, p. 32; Supplementary Methods, lines 524-531; Supporting Information, S1 and S2 File descriptions, p. 15. **Revision:** I clarified the prespecified OR=mean rule wherever isozyme logic is defined, distinguished OR nodes from required AND complexes, and added sensitivity analyses using OR=max and ST6GAL1 alone. The Results, Fig 6, Supplementary Methods, and S2 File now show high overall rank concordance but material changes in some tissues, so local isozyme sensitivity is reported without changing the method post hoc.

Revised text.

> "We used OR=mean as the primary definition and evaluated sensitivity to OR=max and ST6GAL1-only aggregation by recomputing Ng_sia under both alternatives. Tissue rankings were highly concordant (Spearman $\rho = 0.993$ and 0.960, respectively), although OR=max changed individual tissues by as much as 0.580 Z." (lines 528-531)

Reviewer 2 (Konstantinos Flevaris)

R2.1 - Broaden the glycan-biosynthesis modeling literature

Reviewer comment. *The manuscript could provide a broader representation of existing literature on glycan biosynthesis modelling, which would strengthen its scope and contributions to the field.*

Author response. I agree. The original discussion was too narrow and made it difficult to see what reachability adds relative to established modeling strategies. I have added kinetic/ODE, Markov-chain, and automated rule-based glycosylation models and now position reachability at the low-parameter, cross-tissue-comparative end of that spectrum. The revision explicitly presents reachability as complementary to mechanistic models, not as a replacement for them.

Changes in the manuscript. Location: Main manuscript, Introduction, lines 35-47; Discussion Section 4.1, lines 414-447; Table 4, p. 15; the added mechanistic-model references. **Revision:** I added kinetic/ODE, Markov-chain, and automated rule-based glycosylation models, summarized their assumptions and outputs in Table 4, and repositioned reachability as a complementary low-parameter tool for cross-tissue comparison rather than a replacement for mechanistic modeling.

Revised text.

"Glycan biosynthesis has been modeled with kinetic/ODE systems, Markov chains, and rule-based reaction networks; these mechanistic approaches complement transcriptomics-based methods but generally require parameters or reaction rules that are unavailable across many tissues." (lines 36-39)

R2.2 - Same-RNA-seq circularity and an orthogonal validation layer

Reviewer comment. *Because both reachability scores and downstream target scores are derived from the same GTEx RNA-seq data, the analysis does not demonstrate that glycan products mediate the observed associations. It demonstrates transcriptomic coherence between glycosylation-related genes and selected pathway-response genes. This is still valuable, but the language should be calibrated accordingly. Ideally, the authors should include an orthogonal validation layer, such as matched glycomics, glycoproteomics, proteomics, or perturbation data. If this is not feasible, the manuscript should use more precise language that reflects such correlations.*

Author response. I agree with both parts of the comment. First, I adopted the reviewer's term "transcriptomic coherence" and removed language implying glycan-mediated causation. Second, I added an orthogonal HEK293 knockout glycomics benchmark. That experiment provides a perturbation and glycomic readout independent of GTEx, but its interpretation remains deliberately limited: it shows non-random topology-related information while showing no superiority of the minimum over the two prespecified alternatives. The revision therefore supplies the requested orthogonal layer without using it to restore the causal or abundance claims that the same-RNA-seq analysis cannot support.

Changes in the manuscript. Location: Main manuscript, Author Summary, p. 1; Abstract, lines 9-26; Methods Section 2.7, lines 133-193; Results Section 3.7, lines 340-362, and Section 3.9, lines 372-393; Fig 5 and caption, p. 31, and Fig 7 and caption, p. 33; Discussion Section 4.3, lines 465-500; Supplementary Methods, lines 539-629 and 636-642; Supporting Information, S3, S8, S9, and S11 Fig captions, pp. 4, 10, 11, and 13, and S2 Table, p. 14; S2 File. **Revision:** I adopted the term transcriptomic coherence across every affected section, figure, table, and sensitivity analysis and removed causal or validation language from the same-RNA-seq analysis. I then added an orthogonal HEK293 knockout glycomics benchmark, reported it as non-random topology-related information without superiority of the minimum, and kept it separate from any claim that the within-GTEx associations are glycan-mediated.

Revised text.

"These analyses describe the behavior of the constructed score on the same transcriptome and are not an external benchmark of biological accuracy." (lines 358-359)

Revised figure captions.

"Both axes derive from the same RNA-seq and do not establish glycan-mediated signaling." (S3 Fig caption, p. 4)

"Minimum scoring did not outperform mean aggregation or binary topology." (Fig 7 caption, panel A, p. 33)

R2.3 - Bottleneck stability

Reviewer comment. *The bottleneck principle is the central methodological choice. It is useful and interpretable, but a strict minimum can be driven by a single noisy, lowly expressed, or poorly annotated gene. I would encourage the author to report bottleneck stability and/or show whether the same limiting step is repeatedly identified within tissues. This would make the "bottleneck" interpretation more robust.*

Author response. I agree that a bottleneck call should not be interpreted without showing whether it is reproducible within a tissue. I therefore evaluated all 19 multi-step metrics across 50 tissues using per-sample modal frequency, entropy, the gap between the two lowest steps, donor-half splits, and donor bootstrap resampling. This analysis changes the interpretation rather than merely adding diagnostics: pancreas sLeX supports a stable candidate expression-limiting step, whereas brain amygdala GM3 is diffuse, so I no longer assign a confident single-gene brain bottleneck. Stability is now used as a reliability flag, not as proof that the identified gene is biochemically rate limiting.

Changes in the manuscript. Location: Main manuscript, Author Summary, p. 1; Abstract, lines 9-26; Results Section 3.4, lines 276-299; Discussion Section 4.1, lines 414-447, and Section 4.3, lines 465-500; Fig 2D caption, p. 27; Supplementary Methods, lines 630-635; S2 File. **Revision:** I added bottleneck-stability analysis for all 19 multi-step metrics across 50 tissues using modal frequency, entropy, the gap between the two lowest steps, donor-half splits, and donor bootstraps. The Summary, Abstract, Results, Discussion, Fig 2D caption, and complete outputs now distinguish the stable pancreas sLeX call from the diffuse brain amygdala GM3 call and use stability as a reliability flag rather than proof of biochemical rate limitation.

Revised text.

> "Across all 19 multi-step metrics and 50 tissues, the median modal frequency was 0.608, mean donor-half agreement 0.917, and mean bootstrap modal recovery 0.958. We report these quantities as reliability flags and down-weight diffuse, high-entropy calls." (lines 296-299)

R2.4 - Weight of the negative glycomics result

Reviewer comment. *The failed cross-species glycomics comparison and largely negative DepMap essentiality analysis are reported transparently, which is commendable. However, the negative glycomics comparison is especially relevant because direct glycan abundance prediction is the most biologically meaningful external validation. The authors should discuss more explicitly how this affects confidence in the reachability score as a proxy for glycan biosynthetic potential.*

Author response. I agree that the mouse tissue-glycome comparison deserves greater weight than the DepMap analysis because it is the more direct test of an abundance interpretation. The revised Discussion now leads with this null result and states that, together with the brain-ganglioside discrepancy, it lowers confidence that reachability ranks realized abundance across bulk tissues. DepMap is treated as secondary because gene essentiality is less directly connected to the proposed score. I also state what evidence would be needed to revisit the question: matched transcriptomic and glycomic measurements from the same specimens and cell types, followed by perturbation with glycomic readouts. The negative result therefore changes the manuscript's conclusion rather than remaining a peripheral limitation.

Changes in the manuscript. Location: Main manuscript, Author Summary, p. 1; Abstract, lines 9-26; Results Section 3.4, lines 276-299, and Section 3.10, lines 394-412; Discussion Sections 4.2-4.4, lines 448-521; Fig 2B-D and caption, pp. 26-27; Supporting Information, S4, S5, and S8 Fig captions, pp. 5, 6, and 10; S2 File. **Revision:** I moved the null mouse tissue-glycome comparison to the center of the Summary, Abstract, Results, and Discussion; stated that it and the brain-ganglioside discrepancy lower confidence in reachability as a rank of realized bulk-tissue glycan abundance; treated DepMap and the shared-transcriptome glycosidase survey as secondary, non-validating evidence; and specified matched same-specimen transcriptomics/glycomics plus perturbation as the required validation.

Revised text.

> "The mouse tissue-glycome comparison is more consequential and was null. Together with the brain discrepancy, it lowers confidence that reachability is a useful proxy for realized abundance across bulk tissues." (lines 468-470)

Revised figure caption (S5 Fig).

> "None supports a positive abundance association." (S5 Fig caption, p. 6)

S5 Fig reproduced for reviewer convenience. This comparison is shown because its null result materially limits the tissue-level abundance interpretation.

S5 Fig: Cross−species mouse tissue−glycome comparison (Otaki et al. 2022; no significant association)

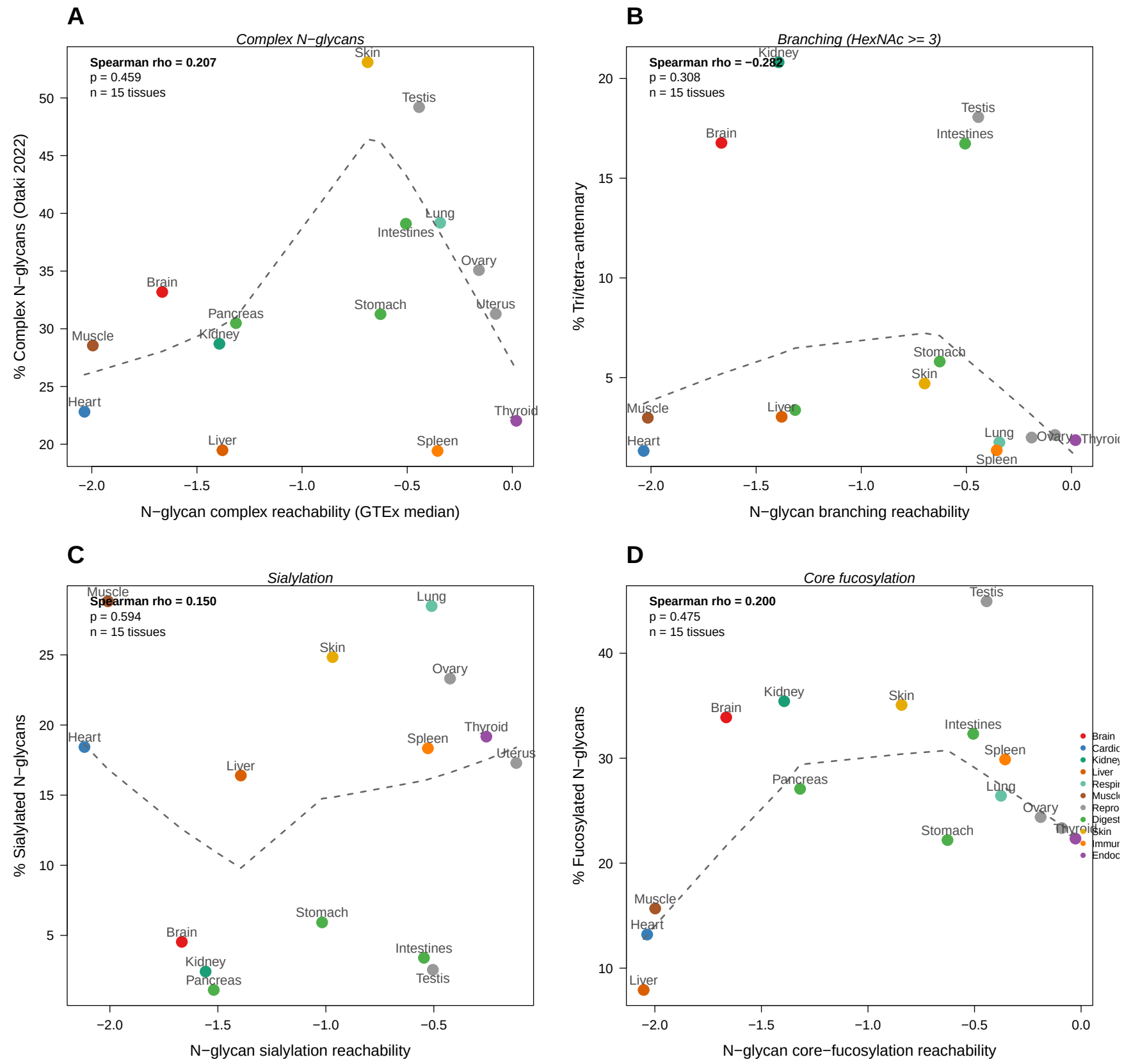

Additional corrections and submission-level changes

The following substantive changes are not assigned to a single reviewer comment. They are listed separately so that the revision record is complete without obscuring the comment-by-comment responses. Pure typesetting, pagination, and PDF-metadata changes are excluded.

Changes in the manuscript. Location: Main manuscript, Methods Section 2.4, lines 107-117; Results Section 3.5, lines 300-313; Fig 3 and caption, pp. 28-29; Discussion Section 4.1, lines 414-447, and Table 4, p. 15; Data and Code Availability, lines 643-655; Financial Disclosure, lines 656-661; Acknowledgments, lines 662-664; Supporting Information inventory, lines 771-790; Supporting Information, S1 and S2 File descriptions, p. 15, and S4 Table caption, p. 16. **Revision:** I corrected the identity of the four tissues excluded by the $n \geq 20$ rule, reconciled the pairwise-test totals and Fig 3C caption with the complete 23-metric analysis, corrected the existing-methods cross-reference to Table 4, expanded the data/code availability statement to identify the versioned reproducibility package, its persistent Zenodo DOI (10.5281/zenodo.21428810), and the complete revision outputs, separated the funding statement from Acknowledgments, added the required funder-role statement, stated that HGA had no specific award number and that Y.M. received salary from HGA, updated the acknowledgments, and reconciled the Supporting Information inventory and file/table descriptions with the submitted materials.

Revised text (sample-inclusion correction).

> "The $n \geq 20$ rule excluded Cervix Ectocervix, Cervix Endocervix, Fallopian Tube, and Kidney Medulla; Bladder ($n = 21$) was retained, yielding 50 tissues. Effect sizes are reported alongside p-values." (lines 114-116)

Revised text (pairwise-statistics correction).

> "Across all 23 metrics, 26,025 of 28,175 tissue-pair tests (92.4%) were significant at within-metric FDR < 0.05. Per-metric proportions ranged from 89.7% (HS 2-O sulfation) to 94.6% (HS polymerization), and median absolute Cliff's delta ranged from 0.537 to 0.900 (Fig 3C). These are descriptive transcriptomic differences; their biological consequence is not established by the tests." (lines 310-313)
