# Supplementary material for: Glycan reachability analysis: A bottleneck-aware framework for inferring tissue-specific glycan biosynthetic potential from transcriptomics
Source: PLoS Comput Biol. 2026 Sep 24;22(9):e1014655. doi: 10.1371/journal.pcbi.1014655 (PMC13606954; doi:10.1371/journal.pcbi.1014655)
Supplement: Peer review history

# Peer review: Decision Letter 0

**DOI:** 10.1371/journal.pcbi.1014655.r001

**Authors:** Pedro Mendes

6 Jul 2026

Glycan Reachability Analysis: A Bottleneck-Aware Framework for Inferring Tissue-Specific Glycan Biosynthetic Potential from Transcriptomics

PLOS Computational Biology

Dear Dr. Matsui,

Thank you for submitting your manuscript to PLOS Computational Biology. After careful consideration, we feel that it has merit but does not fully meet PLOS Computational Biology's publication criteria as it currently stands. Therefore, we invite you to submit a revised version of the manuscript that addresses the points raised during the review process.

Please submit your revised manuscript by Sep 05 2026 11:59PM. If you will need more time than this to complete your revisions, please reply to this message or contact the journal office at ploscompbiol@plos.org. When you're ready to submit your revision, log on to https://www.editorialmanager.com/pcompbiol/ and select the 'Submissions Needing Revision' folder to locate your manuscript file.

Please include the following items when submitting your revised manuscript:

* A letter that responds to each point raised by the editor and reviewer(s). You should upload this letter as a separate file labeled 'Response to Reviewers'. This file does not need to include responses to formatting updates and technical items listed in the 'Journal Requirements' section below.

* A marked-up copy of your manuscript that highlights changes made to the original version. You should upload this as a separate file labeled 'Revised Manuscript with Track Changes'.

* An unmarked version of your revised paper without tracked changes. You should upload this as a separate file labeled 'Manuscript'.

We look forward to receiving your revised manuscript.

Kind regards,

Pedro Mendes

Section Editor

PLOS Computational Biology

**Additional Editor Comments (if provided):**

Thank you for you manuscript on the analysis of glycosylation related transcriptomics. Given the points made by Reviewer 1, particularly regarding the flawed evaluation method, I hesitated between rejection and major-revision. Given the novelty in the approach I am giving you a chance to address those critiques. However, I emphasize that after the next revision I will make an assessment of whether the issue of benchmarking has been addressed appropriately, if not I will have to reject the manuscript. As such, please make sure you carefully address the issue of benchmarking. Obviously, you should also address all other comments from both reviewers.

**Journal Requirements:**

If the reviewer comments include a recommendation to cite specific previously published works, please review and evaluate these publications to determine whether they are relevant and should be cited. There is no requirement to cite these works unless the editor has indicated otherwise.

1) We ask that a manuscript source file is provided at Revision. Please upload your manuscript file as a .doc, .docx, .rtf or .tex. If you are providing a .tex file, please upload it under the item type u2018LaTeX Source Fileu2019 and leave your .pdf version as the item type u2018Manuscriptu2019.

2) Please upload all main figures as separate Figure files in .tif or .eps format. For more information about how to convert and format your figure files please see our guidelines:

https://journals.plos.org/ploscompbiol/s/figures

3) We have noticed that you have uploaded Supporting Information files, but you have not included a list of legends. Please add a full list of legends for your Supporting Information files after the references list.

4) Please amend your detailed Financial Disclosure statement. This is published with the article. It must therefore be completed in full sentences and contain the exact wording you wish to be published.

i) State the initials, alongside each funding source, of each author to receive each grant. For example: "This work was supported by the National Institutes of Health (####### to AM; ###### to CJ) and the National Science Foundation (###### to AM)."

ii) State what role the funders took in the study. If the funders had no role in your study, please state: "The funders had no role in study design, data collection and analysis, decision to publish, or preparation of the manuscript."

iii) If any authors received a salary from any of your funders, please state which authors and which funders..

If you did not receive any funding for this study, please simply state: u201cThe authors received no specific funding for this work.u201d

**Reviewers' comments:**

Reviewer's Responses to Questions

**Comments to the Authors:**

**Please note here if the review is uploaded as an attachment.**

Reviewer #1: The work presented by Yusuku Matsui contains a new computational approach to analyze glycosylation-related transcriptomics data to estimate transcriptomic biosynthetic potential for glycans. Some of the presented algorithmic details are indeed a novel way to approach this kind of data, bringing in more quantitative aspects than the thresholded presenc/absence that many other methods are using. However, the presented work suffers from major methodological uncertainty regarding impact and interpretation, as well as a flawed evaluation/benchmark. I detail some of my concerns in the comments below.

1) The author states "We emphasize that reachability scores measure transcriptomic biosynthetic potential, not enzymatic activity or glycan abundance", yet the entire impact of this article hinges on the relationship to glycan abundance. To illustrate this, the author then contradicts their own limitation later with approaches such as the downstream signaling evaluation, which is implicitly based on assumptions of the impact of the glycans themselves. It is not made evident that there would be genuine applications for "transcriptomic biosynthetic potential" that would not (explicitly or implicitly) be actually targeted to the produced glycan/glycan epitope. Unrealized potential, in other words, does not seem to be of practical interest.

In fact, there are hints in the manuscript that reachability does not reflect glycosylation, such as the unpromising ganglioside reachability assigned to the brain, the region with the highest ganglioside in the body. Similarly, while the transparency is appreciated and acknowledged, the null-finding in the cross-species tissues comparison further indicates that reachability does not yield insights into the produced glycome

2) Related to this, differences of reachability vs other methods are portrayed as "interesting findings" but a mere difference (without method benchmarking etc), especially without founded claims of downstream impacts, carries little information that can be relied upon and should not be overinterpreted

3) Validating the impact of reachability by looking at downstream signaling pathway outputs that are presumably affected by the change in glycosylation is an incredibly indirect and error/confounder-prone way of tackling this problem. Even if such a procedure would be validated/benchmarked (which it is not), it would be highly uncertain. It is also made conceptually impossible by the author's claim that reachability does not indicate produced glycans (which, as stated, are the supposed impact on signaling)

4) All benchmarking that is done in this article (e.g., different aggregation methods) is done with tasks that are themselves not validated, such as the signaling pathway analysis. Targets are being optimized for which the interpretation (and thus the impact/relevance) is very unclear. There is also no data splitting in this, so this essentially presents overfitting

5) Especially for the early results section, it is unclear whether the mere analysis of glycosyltransferase expression across tissues is novel (at the very least it should be placed into context with the many studies that did exactly that, and what they have found)

6) A more general point that the author should at least discuss. Some clear steps, such as core fucosylation & FUT8, demonstrate the potential pitfalls might encounter. For, if FUT8 is knocked out, such as here (https://www.ahajournals.org/doi/10.1161/ATVBAHA.126.324757), core fucosylation can still be detected, just at lower levels. So, ideally, modeling strategies would also model an uncertainty/error term, for unknown enzyme moonlighting activities/compensation, etc

7) Given that reachability scores depend on isozyme means, ST6GAL1/2 may not be the best choice, since ST6GAL2 is barely expressed and will always artificially drag down the workhorse ST6GAL1

Reviewer #2: review attached

**********

**Have the authors made all data and (if applicable) computational code underlying the findings in their manuscript fully available?**

The PLOS Data policy requires authors to make all data and code underlying the findings described in their manuscript fully available without restriction, with rare exception (please refer to the Data Availability Statement in the manuscript PDF file). The data and code should be provided as part of the manuscript or its supporting information, or deposited to a public repository. For example, in addition to summary statistics, the data points behind means, medians and variance measures should be available. If there are restrictions on publicly sharing data or code —e.g. participant privacy or use of data from a third party—those must be specified.

Reviewer #1: Yes

Reviewer #2: None

**********

PLOS authors have the option to publish the peer review history of their article (what does this mean?). If published, this will include your full peer review and any attached files.

If you choose “no”, your identity will remain anonymous but your review may still be made public.

**Do you want your identity to be public for this peer review?**  For information about this choice, including consent withdrawal, please see our Privacy Policy.

Reviewer #1: No

Reviewer #2: **Yes:** Konstantinos Flevaris

[NOTE: If reviewer comments were submitted as an attachment file, they will be attached to this email and accessible via the submission site. Please log into your account, locate the manuscript record, and check for the action link "View Attachments". If this link does not appear, there are no attachment files.]

**Figure resubmission:**

**Reproducibility:**

?>

Attachment Submitted filename: PCOMPBIOL-D-26-00715_feedback.pdf

# Author comment: Author response to Decision Letter 1

**DOI:** 10.1371/journal.pcbi.1014655.r002

20 Jul 2026

Attachment Submitted filename: response_to_reviewers.pdf

# Peer review: Decision Letter 1

**DOI:** 10.1371/journal.pcbi.1014655.r003

**Authors:** Marc Birtwistle

3 Aug 2026

Dear Matsui,

We are pleased to inform you that your manuscript 'Glycan Reachability Analysis: A Bottleneck-Aware Framework for Inferring Tissue-Specific Glycan Biosynthetic Potential from Transcriptomics' has been provisionally accepted for publication in PLOS Computational Biology.

Before your manuscript can be formally accepted you will need to complete some formatting changes, which you will receive in a follow up email. A member of our team will be in touch with a set of requests.

Please note that your manuscript will not be scheduled for publication until you have made the required changes, so a swift response is appreciated.

IMPORTANT: The editorial review process is now complete. PLOS will only permit corrections to spelling, formatting or significant scientific errors from this point onwards. Requests for major changes, or any which affect the scientific understanding of your work, will cause delays to the publication date of your manuscript.

Should you, your institution's press office or the journal office choose to press release your paper, you will automatically be opted out of early publication. We ask that you notify us now if you or your institution is planning to press release the article. All press must be co-ordinated with PLOS.

Thank you again for supporting Open Access publishing; we are looking forward to publishing your work in PLOS Computational Biology.

Best regards,

Marc Birtwistle

Section Editor

PLOS Computational Biology

***********************************************************

Reviewer's Responses to Questions

**Comments to the Authors:**

**Please note here if the review is uploaded as an attachment.**

Reviewer #1: I thank the author for revising their manuscript and responding to my earlier comments via new analyses and text changes/additions, which have both contextualized the work better. While the issue of a relative lack of new insights delivered by this method remains (and it remains unclear whether the method is fundamentally capable of delivering such insights), the performed analyses and discussions might still be of interest to the community and thus might still be useful. I therefore recommend to accept, with the aforementioned caveats

Reviewer #2: The author has adequately addressed my previous comments. I recommend that this manuscript be accepted for publication.

**********

**Have the authors made all data and (if applicable) computational code underlying the findings in their manuscript fully available?**

The PLOS Data policy requires authors to make all data and code underlying the findings described in their manuscript fully available without restriction, with rare exception (please refer to the Data Availability Statement in the manuscript PDF file). The data and code should be provided as part of the manuscript or its supporting information, or deposited to a public repository. For example, in addition to summary statistics, the data points behind means, medians and variance measures should be available. If there are restrictions on publicly sharing data or code —e.g. participant privacy or use of data from a third party—those must be specified.

Reviewer #1: Yes

Reviewer #2: None

**********

PLOS authors have the option to publish the peer review history of their article (what does this mean?). If published, this will include your full peer review and any attached files.

If you choose “no”, your identity will remain anonymous but your review may still be made public.

**Do you want your identity to be public for this peer review?**  For information about this choice, including consent withdrawal, please see our Privacy Policy.

Reviewer #1: No

Reviewer #2: No

# Editor report: Acceptance letter

**DOI:** 10.1371/journal.pcbi.1014655.r004

**Authors:** Marc Birtwistle

PCOMPBIOL-D-26-00715R1

Glycan Reachability Analysis: A Bottleneck-Aware Framework for Inferring Tissue-Specific Glycan Biosynthetic Potential from Transcriptomics

Dear Dr Matsui,

I am pleased to inform you that your manuscript has been formally accepted for publication in PLOS Computational Biology. Your manuscript is now with our production department and you will be notified of the publication date in due course.

The corresponding author will soon be receiving a typeset proof for review, to ensure errors have not been introduced during production. Please review the PDF proof of your manuscript carefully, as this is the last chance to correct any errors. Please note that major changes, or those which affect the scientific understanding of the work, will likely cause delays to the publication date of your manuscript.

Soon after your final files are uploaded, unless you have opted out, the early version of your manuscript will be published online. The date of the early version will be your article's publication date. The final article will be published to the same URL, and all versions of the paper will be accessible to readers.

For Research, Software, and Methods articles, you will receive an invoice from PLOS for your publication fee after your manuscript has reached the completed accept phase. If you receive an email requesting payment before acceptance or for any other service, this may be a phishing scheme. Learn how to identify phishing emails and protect your accounts at https://explore.plos.org/phishing.

Thank you again for supporting PLOS Computational Biology and open-access publishing. We are looking forward to publishing your work!

With kind regards,

Sharmila Kamatchi

PLOS Computational Biology | Carlyle House, Carlyle Road, Cambridge CB4 3DN | United Kingdom ploscompbiol@plos.org | Phone +44 (0) 1223-442824 | ploscompbiol.org | @PLOSCompBiol
